# Exercise protects proliferative muscle satellite cells against exhaustion via the Igfbp7-Akt-mTOR axis

**DOI:** 10.7150/thno.43577

**Published:** 2020-05-16

**Authors:** Zhe Chen, Lei Li, Weiru Wu, Zhilong Liu, Yongxiu Huang, Li Yang, Qing Luo, Jieping Chen, Yu Hou, Guanbin Song

**Affiliations:** 1 Key Laboratory of Biorheological Science and Technology, Ministry of Education, College of Bioengineering, Chongqing University, Chongqing 400044, China.; 2 Department of Hematology, Southwest Hospital, Third Military Medical University (Army Medical University), Chongqing 400038, China.; 3 Clinical hematology, Third Military Medical University (Army Medical University), Chongqing 400038, China.; 4 Medical Research Center, Southwest Hospital, Third Military Medical University (Army Medical University), Chongqing 400038, China.

**Keywords:** Exercise, Muscle satellite cells (SCs), Exhaustion, mTOR, Igfbp7

## Abstract

**Background and Purpose**: The exhaustion of muscle satellite cells (SCs) is correlated with muscle diseases, including sarcopenia and Duchenne muscular dystrophy. Exercise benefits skeletal muscle homeostasis and promotes proliferation of SCs. Elucidating the molecular mechanism underlying the muscle function-improving effect of exercise has important implications in regenerative medicine.

**Methods**: Herein, we investigated the effect of 4-week treadmill training on skeletal muscle and SCs in mice. Hematoxylin and eosin (HE) staining was utilized to detect the morphometry of skeletal muscles. Flow cytometry and immunofluorescence were conducted to analyze the abundance and cell cycle of SCs. RNA sequencing was performed to elucidate the transcriptional regulatory network of SCs. The ChIP-PCR assay was used to detect enrichment of H3K27ac at the promoters of Akt.

**Results**: We observed that exercise resulted in muscle hypertrophy and improved muscle regeneration in mice. Unexpectedly, exercise promoted cell cycling but suppressed the Akt-mTOR pathway in SCs. Proliferative SCs in “exercised mice” required suppressed mTOR activity to limit mitochondrial metabolism, maintaining the “limited activation status” of SCs against exhaustion. Mechanistically, exercise upregulated the expression of Igfbp7, thereby impeding the phosphorylation of Akt and resulting in inhibited mTOR activity and limited mitochondrial metabolism. The limited mitochondrial metabolism resulted in hypoacetylation of histone 3 and reduced enrichment of H3K27ac at promoters of Akt, decreasing the transcription of Akt. Moreover, repeatedly injured mice showed a preserved SC pool and improved muscle regeneration by the suppression of Akt-mTOR signaling.

**Conclusions**: The findings of our study show that exercise protects proliferative SCs against exhaustion via the Igfbp7-Akt-mTOR axis. These findings establish a link between mechanical signaling, mitochondrial metabolism, epigenetic modification, and stem cell fate decisions; thus, present potential therapeutic targets for muscle diseases correlated with SC exhaustion.

## Introduction

Muscle satellite cells (SCs) are a population of muscle stem cells located between the basal lamina and the plasma membrane of muscle fibers. In the homeostatic state, SCs mainly retain quiescence with the expression of Pax7 1. Upon muscle injury or stress, quiescent SCs enter the cell cycle and undergo proliferation and differentiation into myoblasts, forming multinucleated myotubes. Meanwhile, a fraction of activated SCs return to quiescence and replenish the muscle stem cell pool 2. The dysregulated transition of SCs between quiescence and activation can result in SC exhaustion and impair muscle function 2.

The exhaustion of SCs is correlated with several muscle diseases. Sarcopenia is a pathophysiological condition that is commonly observed in elderly individuals and is characterized by low muscle mass, low muscle strength and poor physical performance 3. The decline in the regenerative capacity of skeletal muscles in sarcopenia is due to the functional deterioration of the systemic environment and SC exhaustion 4. Duchenne muscular dystrophy (DMD) is a genetic disorder caused by loss of the protein dystrophin, characterized by severe muscle wasting 5. One widely accepted view is that repetitive cycles of muscle degeneration and regeneration in dystrophic muscles result in eventual exhaustion of SCs 6, 7. Thus, preventing exhaustion of SCs and maintaining SC regenerative potential are potential strategies against sarcopenia and DMD.

Exercise triggers a remodeling program in skeletal muscles that progressively improves muscle mass and strength, and enhances performance in both, young and elderly individuals 8, 9. Physical exercise is the most effective intervention for sarcopenia in elderly individuals 10. Voluntary exercise has also been shown to improve muscle function in DMD mice 11. Exploring the mechanisms underlying this phenomenon and screening exercise analogues are important. Based on microarray analysis of muscle cells, AMP-activated protein kinase (AMPK), and peroxisome proliferator-activator receptor (PPAR) agonists are identified as exercise analogues and are shown to improve muscle performance 12. Exercise induces secretion of irisin from skeletal muscle, and injection of irisin in mice has been shown to induce hypertrophy and to enhance the grip strength of the uninjured muscle 13.

Exercise positively affects muscle fiber composition by regulating SCs to improve muscle performance 14,15. NO-dependent release of hepatocyte growth factor (HGF) induced by exercise, stretch, and loading promotes SC activation and proliferation 16. Growth factor signaling and mechanical loading impinge on the mitogen-activated protein kinase (MAPK) pathway, which functions as an important regulator of proliferation 17. Increased cyclin D1 and p21 mRNA levels in SCs during a resistance-training period may result in the expansion of the SC pool 18. Functional overload induces expression of MyoD in SCs, coinciding with inactivation of Notch signaling and activation of Wnt signaling 19. Exercise, thus, promotes activation of SCs and expands the SC pool. However, whether exercise can maintain the expanded SC pool and how exercise protects proliferative SCs against exhaustion is still unclear.

Herein, in an effort to elucidate the molecular mechanisms underlying the functioning of exercise in the regulation of SCs, we investigated the effect of 4-week treadmill training on skeletal muscles and SCs in mice. Unexpectedly, we found that exercise limited mitochondrial metabolism in SCs to prevent complete activation of SCs. The Igfbp7-Akt-mTOR axis was the key pathway playing a role in the protection of proliferative SCs against exhaustion. Moreover, mice that experienced repeated injuries, suffered severe SC exhaustion, while inhibition of Akt-mTOR signaling showed protective effect on the SC pool and regenerative potential of SCs. Together, our findings demonstrated that exercise protects proliferative SCs against exhaustion via the Igfbp7-Akt-mTOR axis, and this axis may serve as a possible therapeutic target for muscle diseases correlated with SC exhaustion.

## Results

### Exercise promotes muscle hypertrophy and improves muscle regeneration

**Figure 1:**
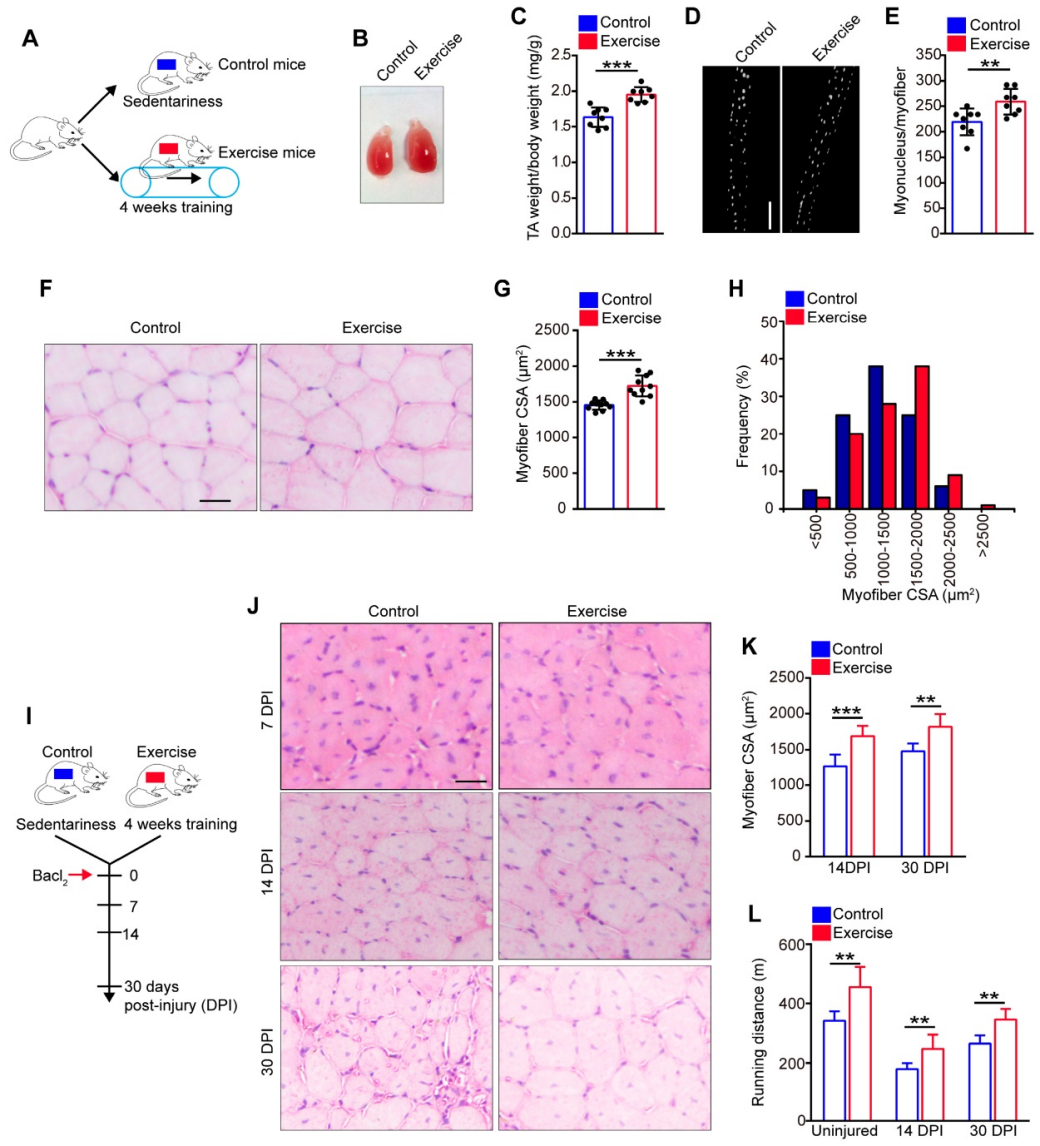
** Exercise promotes muscle hypertrophy and improves muscle regeneration.** (**A**) A schematic illustration showing the experimental design. The mice were designed as “exercise mice” that were subjected to a period of 4 weeks of training (4 days/week, 30 min/day at 12 m/min), while sedentary mice were designed as “control mice”. Skeletal muscles were harvested after 4 weeks of training or sedentariness. (**B**) Visual comparison of the muscle mass of the tibialis anterior (TA) of control and exercise mice. (**C**) TA weight/body weight (mg/g) of control and exercise mice (n=8 mice). (**D**) Immunofluorescence of myonucleus on single myofiber. Nucleus was stained with DAPI. Scale bar, 100 μm. (**E**) Number of myonuclei per myofiber in control and exercise mice (n=8 mice, 20 myofibers per mouse). (**F**) Hematoxylin and eosin (HE) staining of cross-sections of the TA muscle in control and exercise mice. Scale bar, 100 μm. (**G**) Average myofiber cross-sectional area (CSA) of TA in control and exercise mice (n=10 mice). (**H**) Distribution of CSA in control and exercise mice (n=5 mice). (**I**) A schematic illustration showing the experimental design for muscle injury. Bacl_2_ was injected into and along the length of TA and gastrocnemius muscles of mice. The skeletal muscles or TA muscles were harvested at 7-30 days post-injury (DPI) depending on the experiments. (**J**) HE staining of the cross-sections of TA in control and exercise mice at 7 DPI, 14 DPI, and 30 DPI. Scale bar, 100 μm. (**K**) Average CSA of TA in control and exercise mice at 14 DPI and 30 DPI (n=5-10 mice). (**L**) Mice were acclimated to running on the treadmill until exhaustion. The maximum running distances of mice were measured at the indicated time points (n=8 mice). Error bars represent means ± SD. *p<0.05, **p<0.01, ***p<0.001; Student's *t*-test.

**Figure 2:**
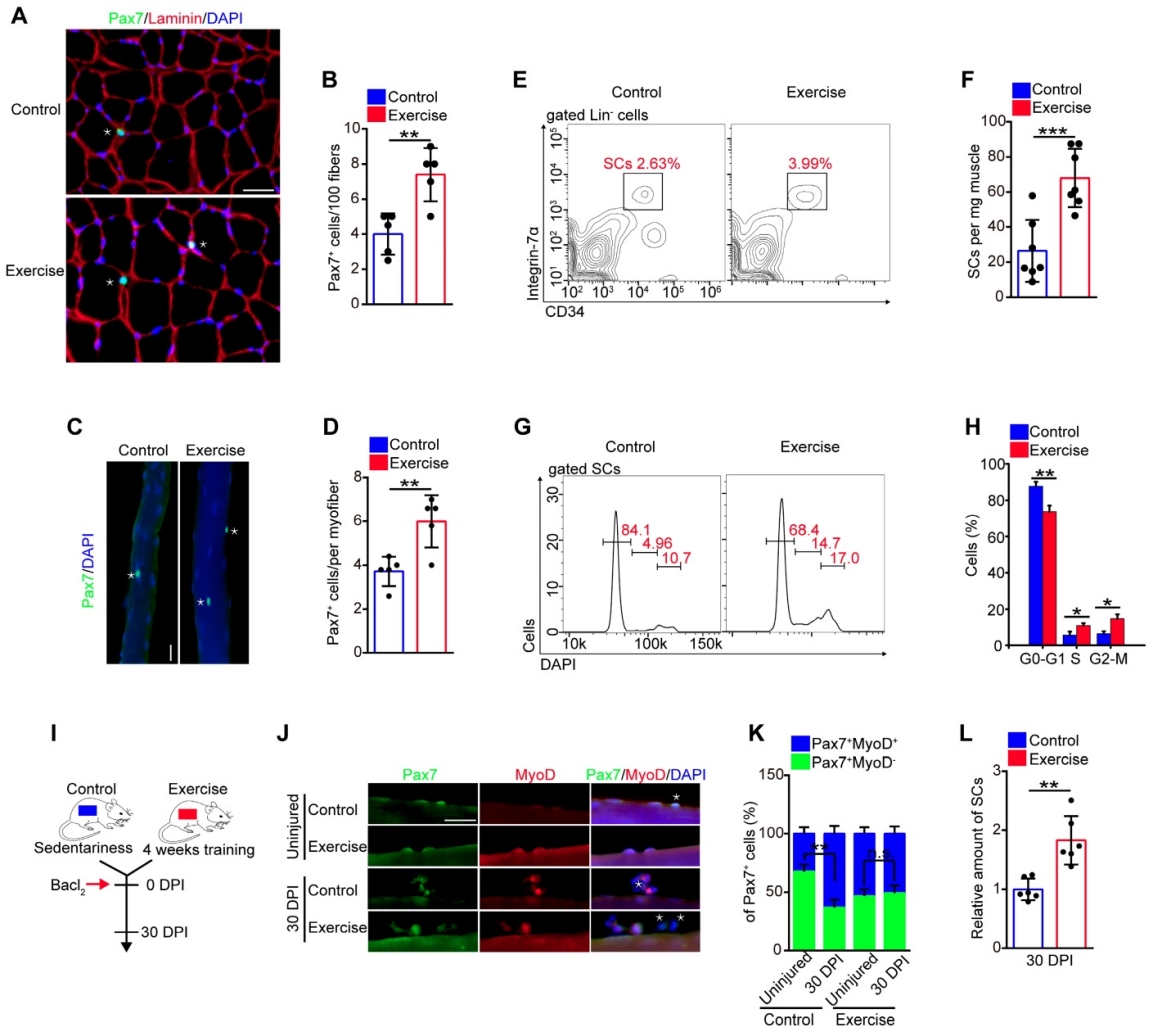
** Exercise promotes SC activation and preserves the expanded SC pool. (A)** Immunofluorescence of Pax7, laminin, and DAPI on cross-sections of TA from control and exercise mice. Scale bars, 100 μm.** (B)** Quantification of the numbers of Pax7^+^ SCs per 100 fibers in cross-sections from control and exercise mice (n=5 mice, 500 fibers per mouse). (**C**) Skeletal muscles were harvested from control and exercise mice. Representative fluorescent images of fresh myofibers immunostained with Pax7 antibody (green) and DAPI (blue). Scale bars, 50 μm. (**D**) Quantification of the numbers of Pax7^+^ SCs per myofiber in control and exercise mice (n=5 mice, 20 myofibers per mouse).** (E)** Skeletal muscles were harvested from control and exercise mice, and then subjected to digestion into mononuclear cells. The cells were stained with cell surface markers (CD45, Sca1,CD11b, CD31, CD34, and α7-integrin). The population of SCs was analyzed by flow cytometry (Lin^-^ cells: CD45^-^Sca1^-^CD11b^-^CD31^-^). (**F**) Quantification of the numbers of SCs per mg muscle of mice (n=7 mice). (**G**) The mononuclear cells isolated from skeletal muscles (after 4 weeks of training or sedentariness) were stained with cell surface markers and DAPI. The cell-cycle of SCs was analyzed by flow cytometry. (**H**) Quantification of the percentage of cell-cycle distribution in SCs (n=5 mice). (**I**) A schematic illustration showing the experimental design for muscle injury. Muscle injury was induced in control and exercise mice by Bacl_2_ injection, and skeletal muscles were harvested before injury or at 30 DPI. (**J**) Representative fluorescent images of fresh myofibers immunostained with Pax7 antibody (green), MyoD antibody (red), and DAPI (blue). Scale bars, 50 μm. (**K**) Quantification of the percentage of quiescent SCs (Pax7^+^MyoD^-^) in Pax7^+^ cells (n=4 mice, 20 myofibers per mouse). (**L**) Skeletal muscles were isolated from mice at 30 DPI and analyzed by flow cytometry. The relative amount of SCs was quantified (n=6 mice). Error bars represent means ± SD. *p<0.05, **p<0.01, ***p<0.001; n.s. no significance; Student's *t*-test.

To explore the precise effect of exercise on skeletal muscle, C57/B6 mice underwent 4 weeks of training on a treadmill (called “exercise mice”), while sedentary mice were defined as “control mice” (**Figure 1A**). The tibialis anterior (TA) was observed to be substantially larger in exercise than control mice (**Figure 1B**). Additionally, the weight of the TA showed a notable increase in exercise mice compared with their control counterparts (**Figure 1C**). Moreover, the myofibers of the extensor digitorum longus (EDL) contained ~25% more myonuclei/myofibers in exercise than control mice (**Figure 2D, E**). Histological analysis of the TA revealed that myofibers were larger in exercise mice compared with their control counterparts (**Figure 1F**). The average myofiber cross-sectional area (CSA) of the TA was significantly larger in exercise than control mice (**Figure 1G**), and the distribution of CSA in exercise mice showed a right shift compared with the control mice (**Figure 1H**). These findings indicated that exercise induced muscle hypertrophy in exercise mice.

To explore the effect of exercise on muscle regeneration, we induced muscle injury by injecting Bacl_2_ into the muscles of mice after 4 weeks of training (**Figure 1I**). Histological analysis revealed larger regenerated myofibers in exercise than control mice at 14 and 30 days post-injury (DPI) (**Figure 1J, K**). We also re-examined exercise performance after injury, and the pre-exercise mice outperformed the control mice in maximum running distance (**Figure 1L**). Together, these data suggested that muscle hypertrophy in exercise mice could be maintained in the injured condition.

### Exercise promotes activation of SCs and preserves the expanded SC pool

We inferred that the muscle hypertrophy in exercise mice might represent an alteration in SCs 10. We observed that the amount of Pax7^+^ SCs in muscle cross-sections in exercise mice was considerably increased compared with their counterparts (**Figure 2A, B**). To substantiate these data, we isolated myofibers and detected SCs by Pax7^+^ staining. The fibers in exercise mice contained more Pax7^+^ SCs than the control mice (**Figure 2C, D**). To further evaluate the SC pool (defined as CD45^-^Sca1^-^CD11b^-^CD31^-^CD34^+^ α7-integrin^+^ cells 1) in exercise mice, we utilized flow cytometry to analyze the SC population. The amount of SCs increased approximately 2.5-fold in exercise mice compared with control mice (**Figure 2E, F**).

The expansion of SCs in exercise mice prompted us to determine the change in cell cycles, apoptosis, and differentiation of SCs, we observed an increased percentage of S phase and G2-M phase in SCs in exercise mice compared with their counterparts (**Figure 2G, H**). Staining of Ki67+DAPI revealed a considerably reduced frequency of quiescent SCs in exercise mice (**Figure S1A, B**). Moreover, the SCs isolated from exercise mice showed more BrdU incorporation than control SCs (**Figure S1C, D**), as well as an elevated proliferation capacity *in vitro* (**Figure S1E, F**). Overload can result in apoptosis of SCs 9; however, the flow cytometry results showed that 4 weeks of training had no obvious effect on SC apoptosis (**Figure S1G, H**). We cultured SCs *in vitro* in differentiation medium and observed a slightly increased numbers of MyHC-positive cells from SCs of exercise mice compared with their counterparts (**Figure S1I, J**). These data demonstrated that exercise promoted SC proliferation and differentiation but did not result in apoptosis.

SCs are mainly retained in a quiescent state, and excessive activation of SCs can result in exhaustion of the SC pool 1,2. To assess whether the activation of SCs induced by exercise might result in SC exhaustion, we examined the abundance of SCs in skeletal muscles *in vivo*. The numbers of Pax7^+^ SCs showed a gradual increase in “trained mice”, compared with sedentary mice, and the increased SC pool was preserved in mice who received continued training (**Figure S2A**). We also detected the amount of SCs after a resting period of 1 or 2 months after exercise (**Figure S2B**). The number of Pax7^+^ SCs still showed an increase in pre-exercise mice compared with control mice (**Figure S2C**). Muscle injury induces activation of SCs and can accelerate the exhaustion of activated SCs. As expected, muscle injury resulted in a reduction of Pax7^+^MyoD^-^ cells (quiescent SCs) in control mice; however, the quiescent SC pool was preserved in exercise mice at 30 DPI (**Figure 2I-K**). Additionally, we induced repeated muscle injuries in mice and observed the preserved number of Pax7^+^ cells in myofibers in exercise mice compared with their counterparts (**Figure S2D, E**). To further strengthen the above results, we performed flow cytometry analysis and demonstrated exercise mice still showed an increased amount of SCs compared with control mice at 30 DPI (**Figure 2L**). These results suggested that exercise promoted SC activation and preserved the expanded SC pool without exhaustion.

### Exercise affects multiple pathways in SCs

**Figure 3:**
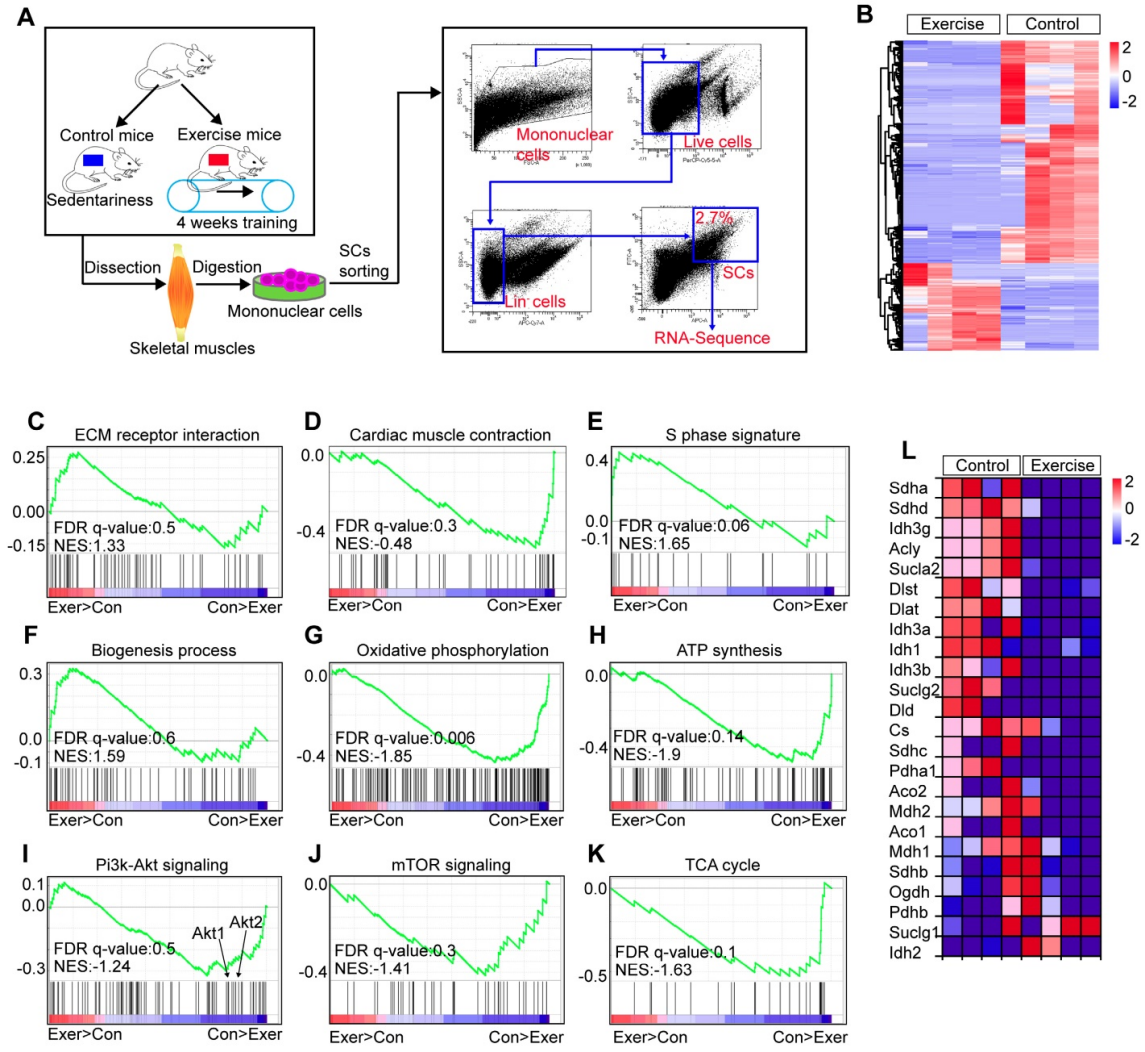
** Exercise affects multiple pathways in SCs.** (**A**) A schematic illustration showing the design used for SC sorting and RNA sequencing. Skeletal muscles were harvested from control and exercise mice and then digested into mononuclear cells. Mononuclear cells were stained with markers, and SCs (defined as Lin^-^CD34^+^ α7-integrin^+^) were sorted by flow cytometry. The sorted SCs were then subjected to RNA sequencing analysis. (**B**) Heatmap of the expression of 1,899 upregulated genes (red) or downregulated genes (blue) by 2-fold or more (*p*<0.01) in SCs (n=4 mice). (**C**-**K**) Gene-set-enrichment analysis (GSEA) of selected gene sets encoding products related to ECM receptor interaction (**C**), Cardiac muscle contraction (**D**), S phase signature (**E**), Biogenesis process (**F**), Oxidative phosphorylation (**G**), ATP synthesis (**H**), Pi3k-Akt signaling (**I**), mTOR signaling (**J**) and TCA cycle (**K**), presented as an enrichment score, as well as genes “positively correlated” with exercise (Exer>Con) or “negatively correlated” with exercise (Con>Exer). Exercise, Exer; Control; Con. (**L**) Heatmap of the expression of TCA cycle-related genes, data from RNA sequencing results.

To explore the molecular mechanisms underlying the effect of exercise on SCs, we performed a transcriptome analysis of SCs in exercise and control mice (**Figure 3A**). We found that 573 genes of SCs in exercise mice were significantly upregulated, while 1,326 genes were significantly downregulated (over 2-fold change, *p*<0.01) (**Figure 3B**). We used gene-set-enrichment analysis (GSEA) to determine whether a pre-defined set of genes showed significantly different expression in SCs. This analysis revealed that the set of genes upregulated in SCs (exercise mice) showed enrichment for the ECM receptor interaction and cardiac muscle contraction pathway (**Figure 3C, D**). Consistent with the observed increased cycling and proliferation of SCs in exercise mice, the set of genes upregulated in SCs showed an enrichment for S phase and biogenesis process (**Figure 3E, F**). Exercise promotes the expression of genes related to mitochondrial metabolism in muscle cells 12,20. However, we found a converse response in muscle satellite cells. Oxidative phosphorylation and ATP synthesis were significantly downregulated in SCs in exercise mice compared with their counterparts (**Figure 3G, H**). Here, enrichment was observed for downregulated gene sets encoding products correlated with Pi3k-Akt signaling, mTOR signaling and the tricarboxylic acid (TCA) cycle (**Figure 3I-K**). Moreover, we found that SCs in exercise mice showed decreased expression of TCA cycle-related genes (**Figure 3L**). Taken together, these results revealed that exercise promoted activation of SCs at the molecular level but suppressed the Pi3k-Akt-mTOR axis.

### Exercise limits mitochondrial metabolism and inhibits Akt-mTOR signaling in SCs

**Figure 4:**
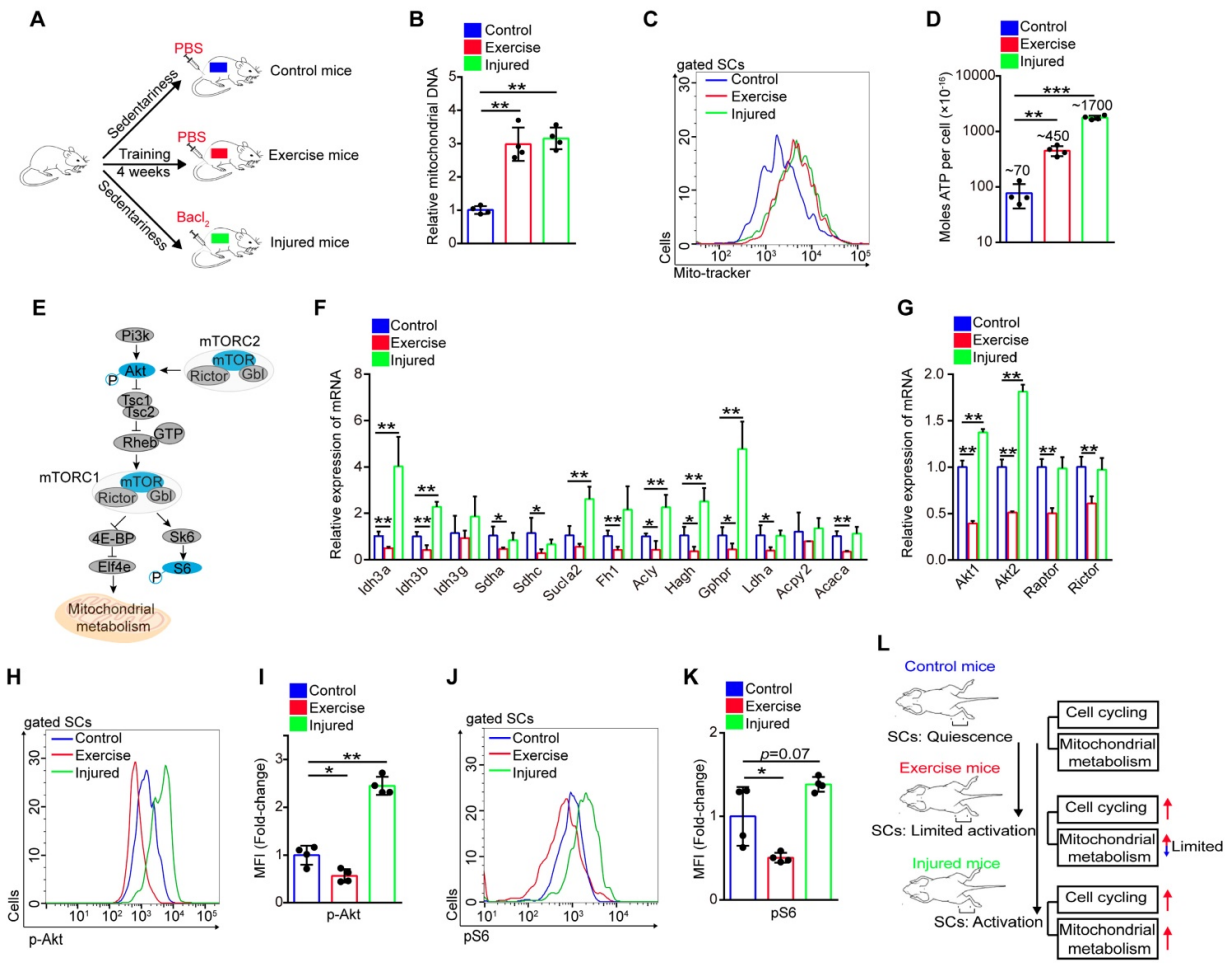
** Exercise limits mitochondrial metabolism and inhibits Akt-mTOR signaling in SCs.** (**A**) A schematic illustration showing the experimental design. Sedentary mice were injected with PBS (defined as “control mice”) or Bacl_2_ (defined as “injured mice”). For experimental precision, “exercise mice” received a PBS injection. Skeletal muscles were harvest at 48 hours after injection of PBS or Bacl_2_. (**B**) SCs were sorted from control, exercise and injured mice. The total DNA of SCs was extracted and the mitochondrial DNA was assessed by qPCR (n=4 mice). (**C**) The mononuclear cells isolated from skeletal muscles of mice were stained with SC markers and mitochondrial fluorescent tracker (Mito-tracker). The intensity of the mito-tracker of SCs was analyzed by flow cytometry. (**D**) SCs were sorted from control, exercise and injured mice. The cellular ATP levels were assayed. (**E**) Summary of the Akt-mTOR pathway. (**F**) Total RNA of sorted SCs was extracted, and the expression of the indicated genes was assayed by qPCR (n=4 mice). (**G**) SCs were sorted from mice. Total RNA of SCs was extracted and the expression of indicated genes was assayed by qPCR (n=4 mice). (**H**) The mononuclear cells isolated from skeletal muscles were stained with SC markers and phosphorylated Akt (p-Akt) fluorescent antibody. The intensity of p-Akt was analyzed by flow cytometry. (**I**) MFI analysis of p-Akt in SCs (n=4 mice). (**J**) The mononuclear cells isolated from skeletal muscles of mice were stained with SC markers and phosphorylated S6 (pS6) fluorescent antibody. The intensity of pS6 was analyzed by flow cytometry. (**K**) MFI analysis of pS6 in SCs (n=4 mice). (**L**) A schematic illustration showing the definition of “limited activation status” of SCs. Error bars represent means ± SD. *p<0.05, **p<0.01; One-way ANOVA.

Exercise promoted SC proliferation, but it seemed to inhibit pathways associated with energy metabolism. Muscle injury is a well-recognized stimulus for SC activation and activated SCs in injured mice show considerably increased mitochondrial metabolism compared with quiescent SCs 21. To assess the exact effect of exercise on the metabolic profile of SCs, we examined the rate of protein biogenesis by measuring the incorporation of the alkyne puromycin analog O-propargyl-puromycin (OP-Puro) into nascent peptides following addition into the culture medium of SCs. SCs from exercise and injured mice incorporated considerably more OP-Puro than control cells *in vitro* (**Figure 4**A and **S3A, B**). We analyzed the mitochondrial content of SCs using qPCR and flow cytometry, and found that the SCs in both exercise and injured mice displayed an increased number of mitochondria (**Figure 4B, C**). Moreover, SCs in exercise and injured mice consumed more glucose than control SCs *in vitro* (**Figure S3C**). Counterintuitively, SCs in exercise mice showed limited ATP levels compared with SCs in injured mice (from ~450×10^-16^ nM/per cell to ~1,700×10^-16^ nM/per cell) but elevated ATP levels compared with SCs in control mice (~70×10^-16^ nM/per cell) (**Figure 4D**). The content of mitochondria, TCA cycle and subsequent oxidative phosphorylation in mitochondria affect cellular ATP production (**Figure 4E**). Here, we discovered that muscle injury promoted the expression of most TCA cycle genes in SCs, but exercise conversely inhibited the expression of such genes compared with their counterparts (**Figure 4F**). Correspondingly, we observed considerable decreases in the activities of respiratory chain complex I and III in the lysate of SCs (unit/mg protein) from exercise compared with control mice (**Figure S3D**). However, the activities of complex I and III per SC (unit/cell) were unaffected compared with their counterparts (**Figure S3E**), which might be attributed to the increased mitochondrial content in SCs. Additionally, we found that exercise promoted expression of the peroxisome proliferator-activator receptor-γ (PPARγ) coactivator-1α (PGC-1α) in SCs (**Figure S3**F), which drives mitochondrial biogenesis in muscle cells. Altogether, these data indicated that both exercise and injury promoted protein biogenesis, mitochondrial synthesis and glucose consumption in SCs, but it inhibited mRNA expression related to oxidative phosphorylation, resulting in limited mitochondrial metabolism and limited cellular ATP production.

Akt-mTOR signaling is a vital upstream pathway of mitochondrial metabolism (**Figure 4E**). Here, we found that the expression of Akt1/2 showed remarkable downregulation in SCs of exercise compared with control mice, but SCs of injured mice showed upregulated expression of Akt1/2 (**Figure 4G**). Phosphorylation of Akt promotes activation of mTOR, and mTOR is crucial for mitochondrial metabolism and phosphorylation of S6, a surrogate of mTORC1 activity 21, 22. Using flow cytometry, we analyzed the intensity of p-Akt and mTOR activity (shown by the intensity of pS6). We found that exercise resulted in decreased phosphorylation of Akt (**Figure 4H, I** and **S3G, H**). The same tendency was found in the activity of mTOR (**Figure 4J, K**). These data demonstrated that exercise inhibited Akt-mTOR signaling in SCs, combined with limited mitochondrial metabolism. Unlike activated SCs in injured mice, we define activated SCs in exercise mice as “limited activation status” (**Figure 4L**).

### Exercise activates MAPK signaling to promote cycling of SCs

Although we found that Akt-mTOR signaling in SCs showed the opposite response in exercise and injured mice, we observed similar changes in several genes. The expression of MyoD (a key transcription factor for SC activation) and cell cycle-related genes (cyclin B1 and cyclin B2) of SCs were elevated in SCs of exercise mice and injured mice (**Figure S4A, B**). The expression of p21 and p27 were downregulated in SCs of exercise and injured mice (**Figure S4B**). We also found that SCs in exercise mice seemed to be less responsive to injury, as the expression of p21, p27, Cyclin B1 and Cyclin B2 showed no obvious change in SCs of injured exercise mice compared with exercise mice (**Figure S4B**).

Activation of MAPK signaling is vital for SC activation and cell cycle entry. Here, we found that both exercise and muscle injury promoted phosphorylation of p38 and ERK *in vitro* and *in vivo* (**Figure S5A-D**). In contrast, prevention of the activation of p38 and ERK evidently abolished the enhanced SC proliferation *in vitro* (**Figure S5E, F**). Notably, blocking the activation of p38 and ERK impeded cell cycling of SCs induced by exercise (**Figure S5G-I**). These results demonstrated that exercise and injury promoted SC cell cycling, partially via the MAPK pathway.

### Exercise protects proliferative SCs against exhaustion by inhibiting Akt-mTOR signaling

**Figure 5:**
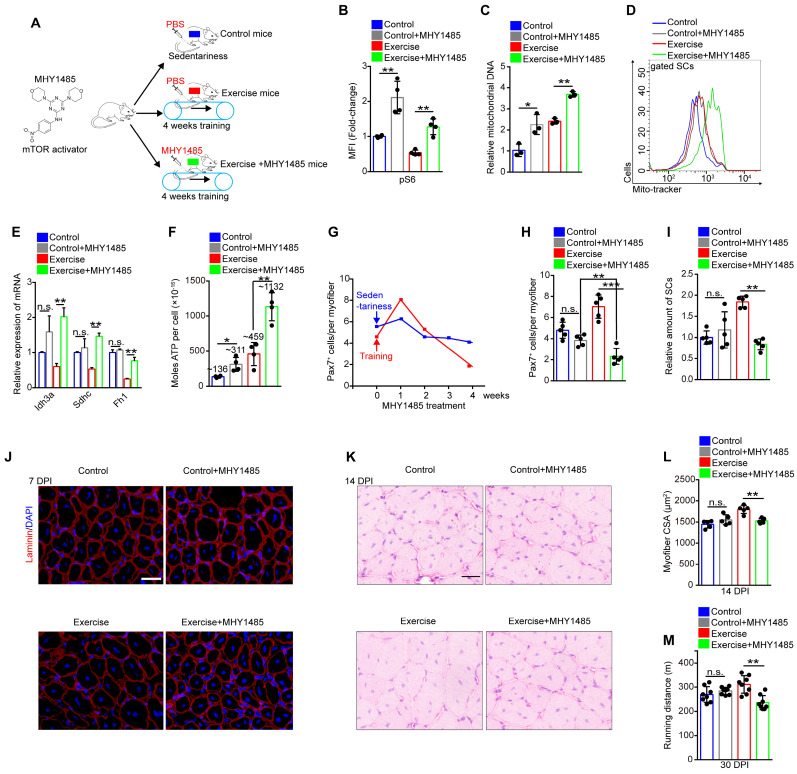
** Exercise protects proliferative SCs against exhaustion by inhibiting Akt-mTOR signaling.** (**A**) A schematic illustration showing the design of the treatment of mTOR activator, MHY1485. Sedentary mice were injected with PBS (defined as “control mice”). Sedentary mice were defined as “control+MHY1485 mice” that were injected with MHY1485 (3 μg/kg, intraperitoneal injection, 4 times/week, 4 weeks). For experimental precision, “exercise mice” received a PBS injection, and mice were defined as “exercise+MHY1485 mice” that were subjected to 4 weeks of training and synchronized MHY1485 injection. (**B**) MFI analysis of pS6 in SCs (n=4 mice). (**C**) SCs were sorted from mice. The total DNA of SCs was extracted, and the mitochondrial DNA was assessed by qPCR (n=3 mice). (**D**) The mito-tracker intensity of SCs was analyzed by flow cytometry (n=3-4 mice). (**E**) SCs were sorted from mice. Total RNA of SCs was extracted, and the expression of indicated genes was assayed by qPCR (n=3 mice). (**F**) SCs were sorted from mice. The cellular ATP level was assayed (n=3-4 mice). (**G**) Mice were subjected to sedentariness or training, as well as synchronized MHY1485 injection. Skeletal muscles were harvested depending on the MHY1485 treatment time (0, 1, 2, 3, and 4 weeks) and digested into single myofibers. The numbers of Pax7^+^ SCs per myofiber were counted (n=3 mice, 20 myofibers per mouse). (**H**) Quantification of the numbers Pax7^+^ SCs per myofiber in mice (n=5 mice, 20 myofibers per mouse). (**I**) The relative amount of SCs per total mononuclear cells isolated from skeletal muscles was quantified (n=5 mice).** (J)** Immunofluorescence of laminin and DAPI on cross-sections of TA from mice at 7 DPI. Scale bars, 100 μm. (**K**) HE staining of the cross-sections of TA in control and exercise mice at 14 DPI. Scale bar, 100 μm. (**L**) Average CSA of TA in control and exercise mice at 14 DPI (n=5 mice). (**M**) Mice were acclimated to running on the treadmill until exhaustion. The maximum running distances of mice were measured at 30 DPI (n=8 mice). Error bars represent means ± SD. *p<0.05, **p<0.01, ***p<0.001; n.s. no significance; One-way ANOVA.

To test whether limited mitochondrial metabolism was associated with the decreased mTOR activity, mice being trained were subjected to synchronized injection of a mTOR activator, MHY1485 (**Figure 5A**). Although the injection of MHY1485 affected all muscle cells, we found that MHY1485 treatment efficiently elevated mTOR activity in SCs, approximately to the level of mTOR activity in control SCs (**Figure 5B**). Enforcing activation of mTOR considerably promoted mitochondrial biogenesis (**Figure 5C, D**) and significantly increased the expression of TCA cycle-related genes (Idh3a, Sdhc, and Fh1) and oxidative phosphorylation genes (**Figure 5E** and** S6A**). The cellular ATP level in SCs was strikingly elevated with MHY1485 treatment (~1132×10^-16^ nM/per cell) compared with exercise mice without MHY1485 treatment (~459×10^-16^ nM/per cell) (**Figure 5F**). These data suggested that exercise limited mitochondrial metabolism of SCs mainly by inhibiting mTOR activity.

It has been reported that the maintenance of hematopoietic and intestinal stem cells require restricted mitochondrial metabolism 23, 24. Therefore, we inferred that exercise inhibited Akt-mTOR signaling and limited mitochondrial metabolism to protect proliferative SCs against exhaustion. Since MHY1485 treatment restored mTOR activity in SCs (**Figure 5B**), we monitored the number of SCs during MHY1485 treatment. MHY1485 treatment for 4 weeks resulted in a slight reduction of the number of Pax7^+^ SCs in sedentary mice (**Figure 5G**). Notably, MHY1485 treatment and synchronized training resulted in increased numbers of Pax7^+^ SCs at 1 week but strikingly decreased numbers of Pax7^+^ SCs at 4 weeks (**Figure 5G**), with no obvious changes in the cell cycle of SCs (**Figure S6B, C**). Compared with control mice, exercise mice were more sensitive to MHY1485 treatment, as shown by the significantly reduced numbers of Pax7^+^ SCs (**Figure 5H**). The flow cytometry results also demonstrated that enforced activation of mTOR signaling completely abolished the expanded SC pool induced by exercise (**Figure 5I**). Moreover, enforced activation of mTOR abolished the enhanced muscle regeneration and performance in exercise mice (**Figure 5J-M**). These data demonstrated that exercise protected proliferative SCs against exhaustion by inhibiting Akt-mTOR signaling.

### Exercise inhibits phosphorylation of Akt by upregulating insulin-like growth factor-binding protein 7 (Igfbp7)

**Figure 6:**
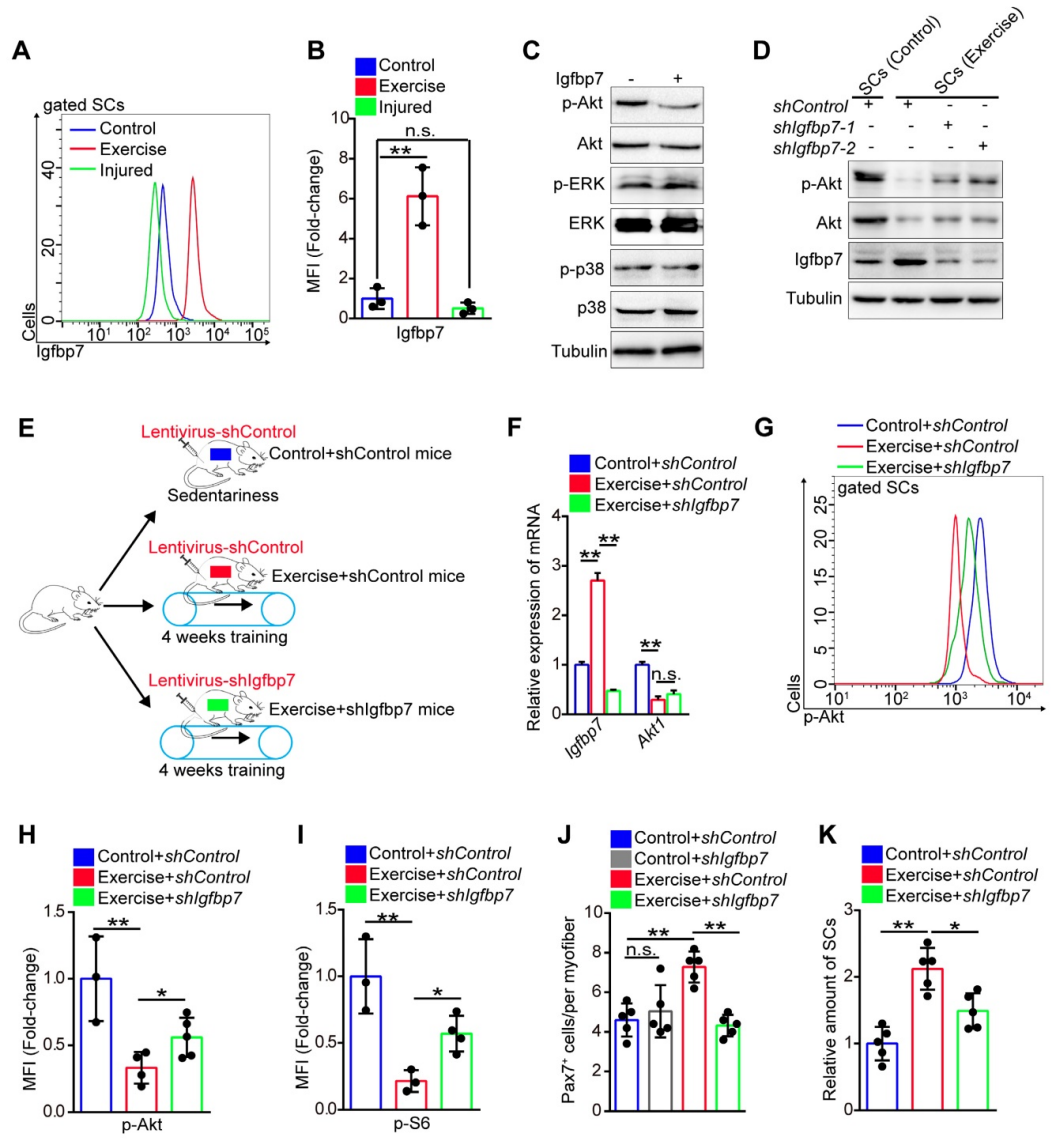
** Exercise inhibits phosphorylation of Akt by upregulating Igfbp7.** (**A**) The mononuclear cells isolated from skeletal muscles of mice were stained with SC markers and Igfbp7 antibody. The intensity of Igfbp7 was analyzed by flow cytometry. (**B**) MFI analysis of Igfbp7 in SCs (n=3 mice). (**C**) SCs of control mice were isolated and cultured in vitro with treatment of Igfbp7 protein (10ug/ml, 12 hours), and the levels of p-Akt, Akt, p-ERK, ERK, p-p38, p38 and Tubulin (loading control) were analyzed by western blot (n=3). (**D**) SCs were isolated and cultured *in vitro*. The SCs were interfered with lentivirus targeting Igfbp7 (*pLKO-shIgfbp7*). Two independent shRNAs were utilized. The protein levels of p-Akt, Akt, Igfbp7 and Tubulin were determined by western blot (n=3). (**E**) A schematic illustration showing the design for the lentivirus treatment targeting Igfbp7. Sedentary mice were infected with lentivirus-shControl (two times infection, defined as “control+shControl”). Mice were defined as “exercise+shControl” that were subjected to 4 weeks of training and synchronized lentivirus-shControl infection, and mice were defined as “exercise+shIgfbp7” that subjected to 4 weeks of training and synchronized lentivirus-shIgfbp7. (**F**) Total RNA of sorted SCs was extracted and the expression of Igfbp7 and Akt1 was assayed by qPCR (n=3). (**G**) The mononuclear cells isolated from skeletal muscles of mice were stained with SC markers and p-Akt antibody. The intensity of p-Akt was analyzed by flow cytometry. (**H**) MFI analysis of p-Akt in SCs (n=3-5 mice). (**I**) MFI analysis of pS6 in SCs (n=3-4 mice). (**J**) Quantification of the numbers of Pax7^+^ SCs per myofiber in mice (n=5 mice, 20 myofibers per mouse). (**K**) The mononuclear cells isolated from skeletal muscles in mice was stained with SCs markers. The relative amount of SCs per total mononuclear cells isolated from the skeletal muscles of mice were quantified (n=5 mice). Error bars represent means ± SD. *p<0.05, **p<0.01; n.s. no significance; One-way ANOVA.

The exercise-induced inhibition of Akt-mTOR signaling in SCs prompted us to screen the key factors regulating Akt. Insulin-like growth factors play an important role in mediating the effect of exercise on humans 25, while Igfbp binds to insulin-like growth factor receptor and blocks its activation 26, 27. Using RNA sequencing, we found that the expression of several Igfbps was evidently downregulated, including Igfbp4, Igfbp5, and Ighbp6 (**Figure S7A-C**). Conversely, the expression of Igfbp7 was considerably elevated in SCs induced by exercise (**Figure S7D**), and the results were confirmed by qPCR (**Figure S7E**) and flow cytometry analysis (**Figure 6A, B**). We found that Igfbp7 protein evidently decreased the phosphorylation of Akt, but it had little effect on the phosphorylation of p38 and ERK *in vitro* (**Figure 6C**). Knockdown of Igfbp7 in SCs partially rescued the phosphorylation intensity of Akt (**Figure 6D**), indicating that exercise might block the phosphorylation of Akt via Igfbp7.

We then utilized the lentivirus to interfere with the expression of Igfbp7 in SCs *in vivo* (**Figure 6E**). Although lentivirus interfered with gene expression of all muscle cells, we found that the expression of Igfbp7 in SCs was significantly knocked down (**Figure 6F**). We discovered that knockdown of Igfbp7 could not effectively rescue Akt1 expression in SCs (**Figure 6F**), but knockdown of Igfbp7 significantly recovered the intensity of p-Akt (**Figure 6G, H**). Subsequently, we found that knockdown of Igfbp7 in SCs partially increased mTOR activity (**Figure 6I**) but negatively affected SC maintenance in exercise mice (**Figure 6J, K**). Moreover, knockdown of Igfbp7 in SCs abolished the enhanced muscle regeneration in exercise mice (**Figure S7F, G**). These data indicated that exercise inhibited Akt-mTOR signaling partially by upregulating Igfbp7, which blocked the phosphorylation of Akt.

### Exercise inhibits transcription of Akt by decreasing H3K27ac

**Figure 7:**
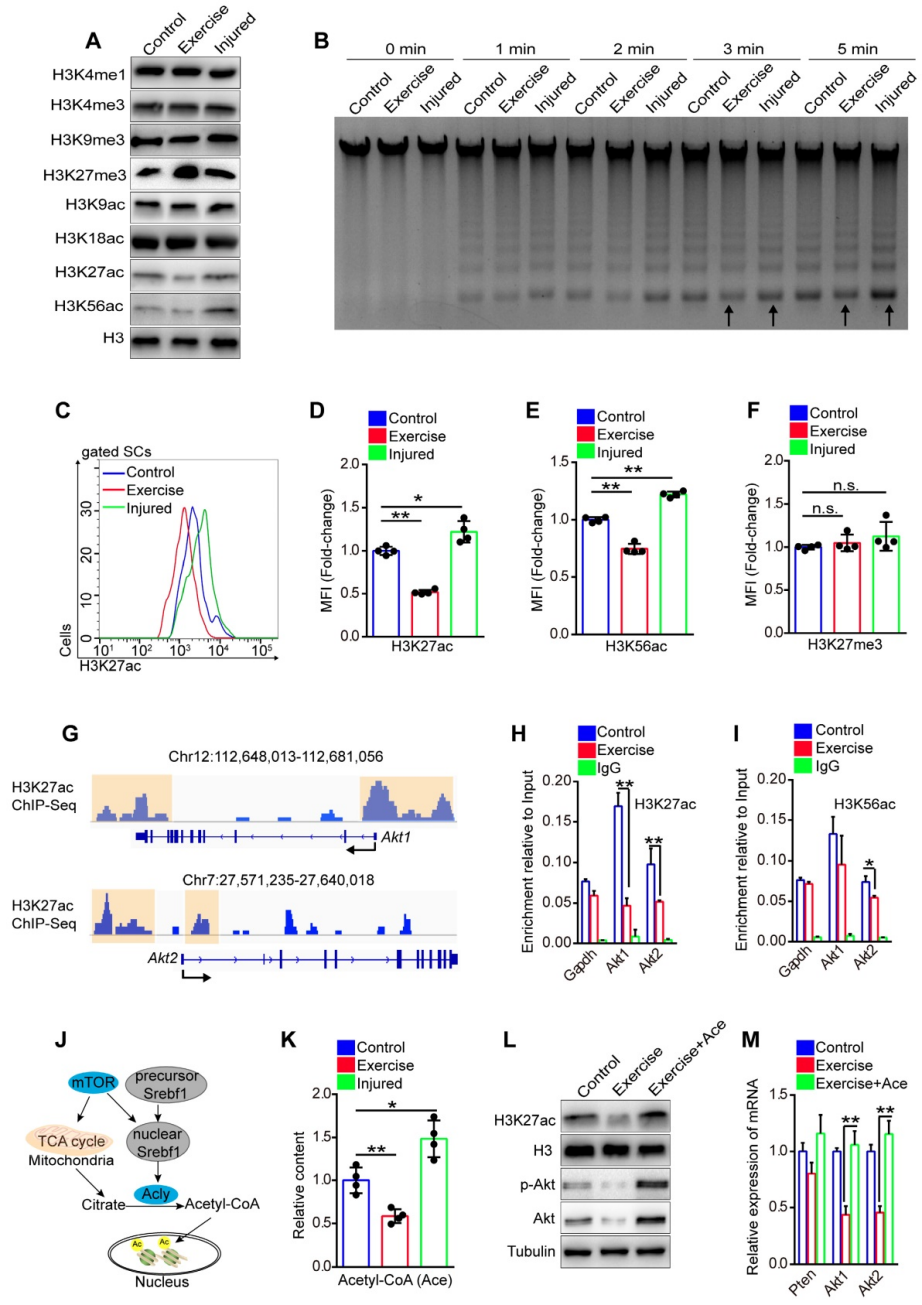
** Exercise inhibits transcription of Akt by decreasing H3K27ac.** (**A**) SCs were isolated from mice and cultured *in vitro*. The levels of modified histone 3 were analyzed by western blot (n=3). (**B**) Cell nucleus was isolated from 0.5 million cultured SCs and adjusted to micrococcal nuclease (MNase) treatment (0.01unit/μl) with increasing durations (0, 1, 2, 3 and 5 min). The DNA fragments were extracted and analyzed by agarose gel electrophoresis (n=3). (**C**) The mononuclear cells isolated from skeletal muscles of mice were stained with SC markers and H3K27ac antibody. The intensity of H3K27ac was analyzed by flow cytometry. (**D**) MFI analysis of H3K27ac in SCs (n=4 mice). (**E**, **F**) MFI analysis of H3K56ac (**E**) and H3K27me3 (**F**) in SCs (n=4 mice). (**G**) H3K27ac ChIP-Sequence signal tracks around the Akt1 and Akt2 genes. The occupancy of H3K27ac was present in promoters of Akt1 and Akt2 in C2C12 cells. Data from GSM3163924. (**H, I**) SCs were isolated and cultured *in vitro*. Quantitative ChIP-PCR was utilized to detect the binding of H3K27ac (**H**) and H3K56ac (**I**) at promoter regions of Akt1/2 in SCs. IgG served as a negative control, and the binding of RNA polymerase II to GAPDH promoter served as a positive control. Enrichment relative to the input was shown (n = 4). (**J**) Summary of the mTOR-histone acetylation pathway. (**K**) SCs were isolated and cultured *in vitro*. The intracellular acetyl-CoA (Ace) levels of SCs were analyzed (n=4). (**L**) SCs were cultured *in vitro* with acetyl-CoA (10 μM) treatment for 24 hours. The protein levels of H3K27ac, H3 (loading control), p-Akt, Akt and Tubulin (loading control) were analyzed by western blot (n=3). (**M**) Total RNA of cultured SCs was extracted and the expression of Pten, Akt1, and Akt2 was assayed by qPCR (n=4). Error bars represent means ± SD. *p<0.05, **p<0.01; n.s. no significance; One-way ANOVA.

Igfbp7 blocked phosphorylation of Akt and had no obvious effect on the transcription of Akt, implying that the inhibited effect of exercise on Akt was not fully elucidated. Exercise alters epigenetic modification of cells, and histone modifications are closely related to gene expression 28. To test whether exercise affected SC epigenetic modification, we detected the modification of histone 3 (H3) in SCs *in vitro*. We found that the intensity of H3K27me3 (triple methylated H3K27), H3K27ac (acetylated H3K27) and H3K56ac (acetylated H3K56) in SCs isolated from exercise mice showed marked differences compared with control and injured mice (**Figure 7A**). The transcription of genes requires accessible chromatin, which is tightly regulated by histone modification 29. Through MNase analysis, we found that chromosomes in SCs from exercise mice had lower accessibility than those from injured mice *in vitro* (**Figure 7B**), confirming the changed H3 modification in SCs of exercise mice. Next, we utilized flow cytometry to detect the H3 modification in SCs *in vivo* and we found that the intensity of H3K27ac and H3K56ac was considerably decreased in SCs of exercise mice, while muscle injury elevated the intensity of H3K27ac and H3K56ac in SCs (**Figure 7C-E**). The intensity of H3K27me3 in SCs of exercise mice was comparable to the control and injured mice (**Figure 7F**).

H3K27ac marks both active promoters and distal enhancers, which are critical for gene expression 30. We analyzed the published data for the H3K27ac ChIP-sequence in the C2C12 cell line, a murine myoblast cell line 31. We discovered H3K27ac peaks in promoter regions of Akt1 and Akt2 (**Figure 7G**), implying a regulatory effect of H3K27ac on Akt1/2 transcription. Next, we conducted a ChIP-PCR assay and demonstrated the occupancy of H3K27ac, H3K56ac, and H3K4me3 at promoter regions of Akt1 and Akt2 in SCs (**Figure S8A-E**). More importantly, we found that SCs from exercise mice showed significantly decreased occupancy of H3K27ac at both promoter regions of Akt1 and Akt2 compared with their counterparts (**Figure 7H**), but the occupancy of H3K56ac and H3K27me3 showed no obvious changes (**Figure 7I and S8F**). These data indicated that the downregulated transcription of Akt in SCs in exercise mice could be attributed to the decreased H3K27ac.

Histone acetylation relies on the substrate, nuclear acetyl-CoA, which is converted by ATP citrate lyase (Acly) utilizing mitochondrial citrate. Citrate is a product of the TCA cycle in mitochondria (**Figure 7J**) 32, 33. To test whether exercise resulted in a reduction of H3K27ac via the limited mitochondrial metabolism, we detected the content of acetyl-CoA in SCs *in vitro*. The results revealed that SCs from exercise mice had the lowest content of acetyl-CoA compared with those from injured or control mice (**Figure 7K**). We supplied acetyl-CoA in the culture medium and found that the acetyl-CoA treatment dramatically rescued the intensity of H3K27ac in SCs of exercise mice (**Figure 7L**). Moreover, the mRNA and protein levels of Akt were considerably increased in the SCs (**Figure 7L, M**). To substantiate these data, we treated SCs with MHY1485 and found that enforcing activation of mTOR effectively recovered the content of acetyl-CoA in SCs from exercise mice (**Figure S9A**). Correspondingly, MHY1485 restored the intensity of H3K27ac (**Figure S9B**) and rescued the occupancy of H3K27ac at the promoters of Akt1/2 (**Figure S9C**). Taken together, these data revealed that exercise reduced the accumulation of acetyl-CoA in SCs mainly via the mTOR pathway, resulting in decreased H3K27ac level and downregulated transcription of Akt.

### Prevention of Akt-mTOR signaling activation preserves the SC pool under stress

**Figure 8:**
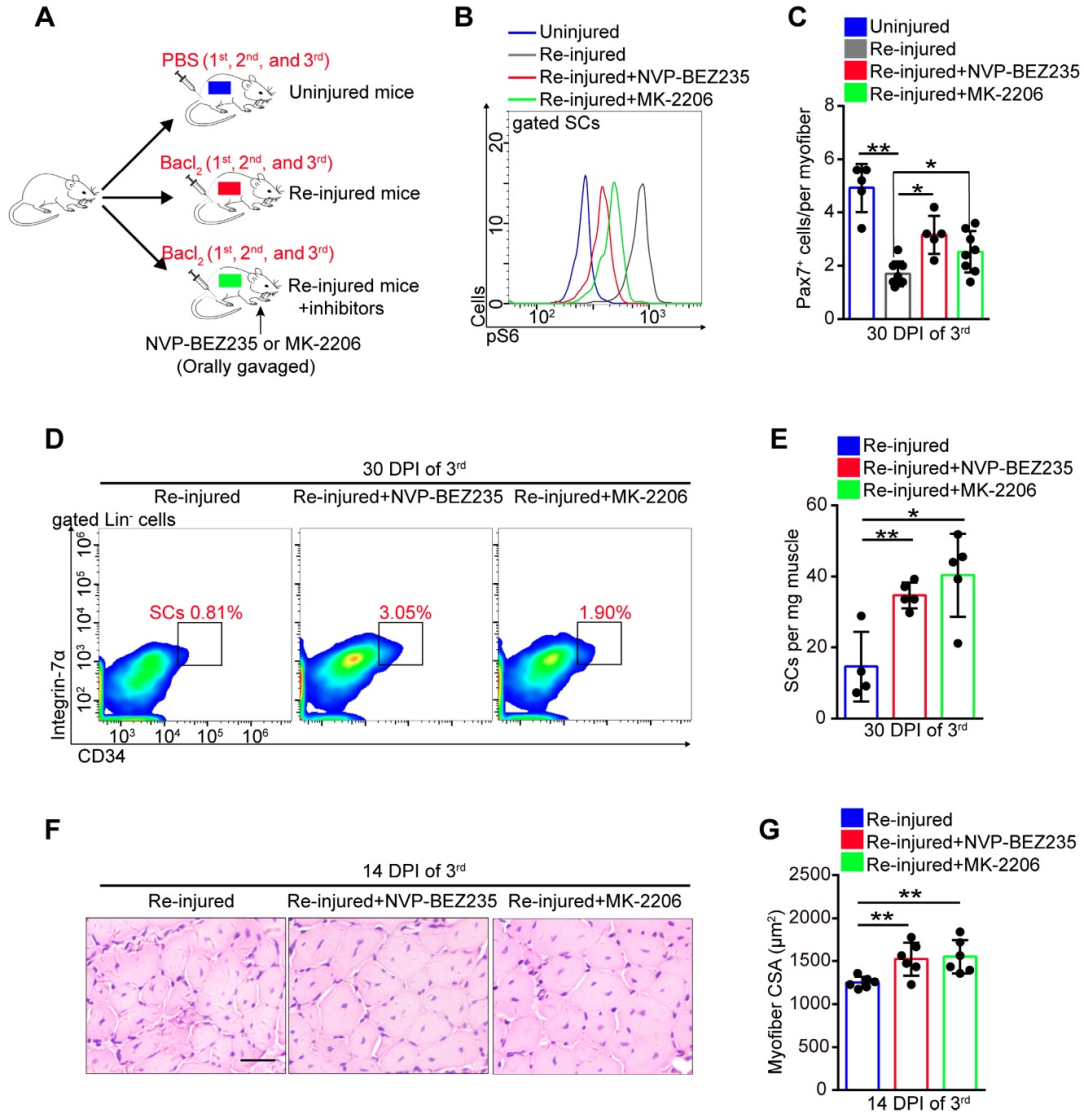
** Prevention of Akt-mTOR signaling activation preserves the SC pool under stress. (A)** A schematic illustration showing the design of repeated muscle injuries. Mice were defined as “uninjured mice” that were injected with PBS (three times, 14-days interval), and mice were defined as “repeated injured mice” (re-injured mice) that were injected with Bacl_2_ (three times, 14-days interval, 1^st^, 2^nd^ and 3^rd^). Mice were defined as “Re-injured+inhibitors mice” that injected with Bacl_2_ and synchronized inhibitor treatment (NVP-BEZ235 or MK-2206). (**B**) The mononuclear cells isolated from skeletal muscle in mice (48 hours after 3^rd^ Bacl_2_ injection) were stained with SCs markers and pS6 antibody. The intensity of pS6 in SCs was analyzed by flow cytometry. (**C**) Quantification of Pax7^+^ SCs number per myofiber of mice at 30 days post-injury (3^rd^ ) (n=5-8 mice, 20 myofibers per mouse). (**D**) The mononuclear cells isolated from skeletal muscles of mice at 30 DPI (3^rd^) were stained with SC markers. The population of SCs was analyzed by flow cytometry. (**E**) Quantification of the numbers of SCs per mg muscle of mice at 30 DPI of the 3^rd^ injury (n=4-5 mice). (**F**) HE staining of the cross-sections of TA in mice at 14 DPI of the 3^rd^ injury. Scale bar, 100 μm. (**G**) Average CSA of TA in mice at 14 DPI of the 3^rd^ injury (n=6 mice). *p<0.05, **p<0.01; n.s. no significance; One-way ANOVA.

Exercise promoted SC proliferation without exhaustion by inhibiting Akt-mTOR signaling. We speculated that inhibitors of Akt-mTOR signaling could serve as exercise analogs. Repeated muscle injuries induce excessive activation of SCs and result in exhaustion of SCs. To test the protective effect of exercise analogs on SCs, we treated repeatedly injured mice with NVP-BEZ235 or MK-2206, which are inhibitors of Pi3k-mTOR and Akt, respectively (**Figure 8A**). NVP-BEZ235 and MK-2206 effectively induced a partial inhibition of mTOR activity in SCs of repeatedly injured mice (**Figure 8B**). In the absence of inhibitor treatment, repeatedly injured mice showed a considerably decreased number of Pax7^+^ SCs compared with uninjured mice (**Figure 8C**). Additionally, mice exposed to triple injuries showed a preserved SC pool with NVP-BEZ235 or MK-2206 treatment (**Figure 8C-E**). Moreover, they also showed improved muscle regeneration upon treatment with NVP-BEZ235 or MK-2206 (**Figure S10A** and **8F, G**), as well as enhanced endurance performance (**Figure S10B**). These data implied that preventing the activation of Akt-mTOR signaling in SCs might be an effective strategy to preserve the SC pool under stress.

## Discussion

A unique property of SCs is their ability to exist in a non-cycling, quiescent state 1, 2. In response to stimuli or damage, quiescent SCs translate into activated SCs, enter the cell cycle, and produce fusion-competent myoblasts to support muscle growth and regeneration 34. The return of activated SCs to quiescence is crucial for SC self-renewal and maintenance. Dysregulation of extracellular and intracellular signaling can lead to excessive activation of SCs, resulting in eventual SC exhaustion 35, 36, such as that observed in sarcopenia and DMD 4, 7. Muscle injury induces SC rapid activation due to the demand for muscle regeneration, and a fraction of SCs return to quiescence and replenish the SC pool 21. Repeated muscle injuries lead to repetitive cycles of SC activation and quiescence, resulting in the eventual loss of the SC pool 37. Thus, the prevention of proliferative SCs against exhaustion has important implications in regenerative medicines, as well as against muscle diseases correlated with SC exhaustion.

Exercise acts as a mechanical stimulus and induces SC activation 38, 39. In this study, we found that exercise significantly promoted SC cell cycling (**Figure 2H**), and the expanded SC pool could be preserved for a long time (**Figure S2A, C**). It is clear that exercise induces SC activation and protects proliferative SCs against exhaustion. The differences in transcriptional profiling of SCs between uninjured and injured mice have been reported, and the mitochondrial metabolism pathway shows upregulated enrichment in SCs of injured mice 21, 40. Moreover, transcriptional profiling in muscle cells of mice has also demonstrated that exercise promotes the upregulation of genes related to mitochondrial metabolism 12. Here, we reported the transcriptional profiling of SCs in exercise mice by RNA sequencing. Unexpectedly, we found that exercise inhibited the expression of mitochondrial metabolism-related genes (TCA cycle and oxidative phosphorylation) (**Figure 3G, K**). Muscle SCs undergo transcriptomic alterations during isolation; thus, the transcriptomic changes in SCs in their native state require further study. Muscle injury promotes SCs activation with increased expression of genes related to the TCA cycle 21, 41, but exercise inhibited the expression of such genes in SCs (**Figure 4F**). Although exercise inhibited the expression of OxPhos genes, it promoted protein synthesis and glucose consumption, resulting in an increased mitochondria content in SCs. Thus, we found that the activities of complex I and III per SC (unit/cell) were unaffected compared with their counterparts, and an elevated ATP level per SC in exercise mice. The mitochondrial content was even comparable between SCs of exercise mice and activated SCs of injured mice (**Figure 4B, C**), the cellular ATP levels were lower in SCs of exercise than injured mice (**Figure 4D**). Our results indicated that exercise limited mitochondrial metabolism of activated SCs in exercise mice, but whether these phenomena exist in humans requires future studies.

Mitochondrial metabolism requires activated Akt-mTOR signaling 42. mTOR forms 2 distinct complexes, namely, mTOR complex 1 (mTORC1) and mTOR complex 2 (mTORC2). mTORC1 controls mitochondrial activity and biogenesis by 4E-BP dependent translationally regulating the expression of nucleus-encoded mitochondrial-related mRNAs, eventually coordinating energy consumption and proliferation 43, 44. It has been reported that muscle injury promotes activation of Akt-mTOR signaling in SCs 21, 40. Here, we confirmed previous findings and observed that exercise inhibited transcription of Akt, phosphorylation of Akt and mTOR activity in SCs (**Figure 4G-K**). Our results suggested that activated SCs in exercise mice were largely different from activated SCs in injured mice. Other researchers have reported that a mechanical stimulus upregulated transcription and phosphorylation of Akt in muscle cells 45, 46, which indicated that SCs and muscle cells had different responses to exercise.

The observation of contralateral SCs (CSCs) induced by distant injury showed that the quiescent status of SCs is composed of two distinct functional phases, namely, G0 and an alert phase. SCs in the alert phase remain dormant but have larger cell sizes and upregulated mitochondrial metabolism than quiescent SCs 21. Here, we found that, similar to activated SCs in injured mice, SCs in exercise mice showed accelerated cell cycling. However, exercise inhibited Akt-mTOR signaling and limited mitochondrial metabolism in SCs. Thus, we proposed that the activated status of SCs was composed of two distinct functional phases, namely, activation and limited activation (**Figure 4L**). Whether other stem cells have a limited activation status warrants further investigation.

The limited activation status of SCs showed suppressed mTOR activity and limited mitochondrial metabolism. The mTOR signaling plays critical roles in stem cell self-renewal and differentiation. It has been reported that hyperactivity of mTOR signaling promotes differentiation and can result in stem cell exhaustion, such as germline stem cells, hematopoietic stem cells and stem cells in the tracheal epithelium 22, 47, 48. Conversely, mTOR inhibits mesendodermal differentiation in human pluripotent stem cells 49. Here, we found that training promoted the early expansion of the SC pool in mice with enforced activation of mTOR (utilizing MHY1485), but eventually resulted in a reduction of SC numbers (**Figure 5G**). Compared with the SCs of sedentary mice, SCs of exercise mice were more sensitive to MHY1485 treatment (**Figure 5H**). mTOR activity is crucial for mitochondrial metabolism, while stem cell maintenance requires restricted mitochondrial metabolism, such as hematopoietic stem cells 22. These results revealed that SCs in exercise mice required suppressed mTOR signaling to maintain the expanded SC pool, which might be an adaptive strategy for SCs against exhaustion upon exposure to long-term exercise.

The phosphorylated Akt activated mTOR via the TSC-Rbeb axis 50, and the decreased mTOR activity in SCs in exercise mice could be attributed to reduced total Akt and phosphorylated Akt. SCs in Akt1-deficient mice still show greater numbers of activated SCs (BrdU^+^Pax7^+^ SCs) after training compared with the control 51, suggesting that exercise promotes SC activation independent of Akt1. Consistently, we demonstrated that exercise promoted SC enter into the cell cycle, which mainly depended on MAPK signaling. Insulin and Igfbps play important roles in the activation of Akt and MAPK 26, 27. Generally, Igfbps bind insulin-like growth factor receptor and block its activation 27, 50. Here, we discovered that exercise downregulated the expression of Igfbp4, Igfbp5, and Igfbp6 in SCs of exercise mice, but considerably upregulated the expression of Igfbp7 (**Figure 6B**), suggesting that Igfbp7 might be a special mediator of the exercise-impeding activation of Akt (phosphorylated Akt). Indeed, knockdown of Igfbp7 in SCs partially recovered the intensity of p-Akt and mTOR activity (**Figure 6H, I**), resulting in that the expanded SCs pool in exercise mice could not be maintained (**Figure 6J**). The roles of Igfbp3 and Igfbp5 in myogenesis have been reported 53, 54. Our findings uncovered the role of Igfbp7 in protecting proliferative SCs of exercise mice, but whether Igfbp7 regulates the maintenance of quiescent SCs and affects muscle homeostasis remains unknown.

The expression of total Akt could not be effectively rescued by knockdown of Igfbp7 (**Figure 6F**), implying that other factors inhibited the transcription of Akt. Here, we found that exercise resulted in histone hypoacetylation in SCs predominantly at lysine 27 and 56 of histone 3 (H3K27 and H3K56) (**Figure 7C-E**), which promotes gene expression and chromatin accessibility 55. Moreover, we demonstrated that the transcriptional downregulation of Akt was attributed to a reduced occupancy of H3K27ac at promoter regions of Akt (**Figure 7H**). Histone acetylation relies on the substrate, nuclear acetyl-CoA, which is converted by Acly utilizing mitochondrial citrate 29, 20. Acly promotes SC differentiation by altering histone acetylation (H3K9ac, H3K14ac, and H3K27ac) 56. We found that exercise reduced substrate accumulation for histone acetylation in SCs (**Figure 7K**) and we observed that supplementation with acetyl-CoA efficiently recovered the transcription of Akt (**Figure 7L, M**). These results uncovered the underlying mechanisms responsible for exercise-induced inhibition of Akt activation at the transcriptional and post-translational levels.

The balance of Pi3k-Akt-mTOR signaling is critical for maintenance of the SC pool. Deletion of p110α, a catalytic subunit of phosphatidylinositol 3-kinase (Pi3k), resulted in SCs unable to exit quiescence. However, the induction of a constitutively active Pi3k in quiescent SCs resulted in spontaneous SC activation and eventual exhaustion of the SC pool 57. Here, we found that serial muscle injuries led to activation of mTOR signaling and a reduced SC pool. Through interference in Pi3k-Akt-mTOR signaling, SCs in mice with repeated injuries were effectively preserved and showed enhanced regenerative potential. Moreover, inhibition of mTOR signaling via rapamycin ameliorates the dystrophic phenotype in the skeletal muscle of MDX mice 58. Thus, inhibition of Pi3k-Akt-mTOR signaling might be an effective strategy to protect SCs against exhaustion under stress.

In conclusion, our study demonstrated that exercise protected proliferative SCs against exhaustion via the Igfbp7-Akt-mTOR axis by limiting mitochondrial metabolism and maintaining the limited activation status of SCs. These findings establish a link between mechanical signaling, mitochondrial metabolism, epigenetic modification, and stem cell fate decisions, revealing potential therapeutic targets for muscle diseases correlated with SC exhaustion.

## Methods

**Exercise training and drug treatment.** Male C57B/6J mice (10 weeks old) were randomly divided into two groups (1) sedentary (control mice) and (2) treadmill running (exercise mice). The mice in the exercise mice were subjected to 4 weeks of running exercise (4 days/week, 30 min/day at 12 m/min) on a treadmill (ZH-PT, Zheng Hua) at 5°. For the treadmill test, mice in all groups were acclimated to moderate treadmill running (10 m/min for 5 min daily) for 3 consecutive days before the test. Then mice ran on the treadmill at 10 m/min for 5 min, and the speed was increased from 10 to 16 m/min and then maintained constant until exhaustion. During the 4 weeks of training, mice from exercise mice were injected intraperitoneally (the same frequency with training) with SB203580 (15 mg/kg, Selleck) and FR 180204 (10 mg/kg, Selleck), or MHY1485 (3 μg/kg, Selleck). During muscle serial injury, mice were orally gavaged with NVP-BEZ235 (40mg/kg, Selleck) or MK-2206 (10mg/kg, Selleck). All mice experiments were approved by the Animal Ethics Committee of Chongqing University.

**Skeletal muscle injury**. To induce injury, 50 ul 1.2% BaCl_2_ in PBS or PBS alone was injected into and along the length of tibialis anterior (TA) and gastrocnemius muscles 59. The skeletal muscles or TA muscles were harvested 2-30 days after injury depending on the experiments.

**Histology and morphometric analysis**. TA muscles were fixed for 12 hours using 4% paraformaldehyde and subsequently transferred to 20% sucrose overnight. For the assessment of muscle morphology, 10 μm thick transverse sections of TA muscle were subjected to haematoxylin and eosin (HE) staining or immunofluorescence. For quantitative analysis, the cross-sectional area (CSA) was performed using AxioVision software (Zeiss) with a minimum of 500 fibers. For injured mice, only the CSA of regenerating/centrally-nucleated fibers were quantified.

**Isolation of single myofibers**. Single myofibers were isolated from extensor digitorum longus (EDL) muscles and digested in Dulbecco's modified Eagle's medium (DMEM) (catalogue number: SH30022.01, Hyclone, GE, USA) with 0.2% collagenase (Sigma-Aldrich, USA) at 37ºC for 90 min. Fibers were liberated by trituration in DMEM medium with Pasteur pipettes.

**Satellite cell isolation and culture.** To culture SCs in vitro, we obtained SCs using single myofiber cultures. Briefly, single fibers were placed in Matrigel-coated dishes (BD Bioscience, USA) in fiber medium consisting of DMEM (Hyclone) with 20% fetal bovine serum (Hyclone), 1% penicillin/streptomycin (Hyclone), and 1% Chick embryo extract (US biological, USA) at 37ºC with 5% CO_2_. SCs migrated off the myofibers in 3 to 4 days. To analyze the growth of SCs, isolated SCs were cultured in fiber medium. For differentiation, SCs were cultured in DMEM containing 2% horse serum (Gibco, Thermo Fisher, USA) on Matrigel. For the supply of substrate for histone acetylation, SCs were treated with 10uM acetyl-CoA (Sigma) for 24 hours before analysis. For Igfbp7 treatment, recombinant full-length Igfbp7 proteins (10ug/ml, Abcam) were added to the medium for 12 hours.

**Flow cytometry**. Satellite cell (SCs) isolation and purification was performed according to established methods. Briefly, tibialis anterior muscle of mice was subjected to 0.2% collagenase (Sigma) for 90 min and then 0.2% dispase (Sigma) for 30 min. The cell suspension was filtered through a 70 μm nylon filter (Falcon) and mononuclear cells were collected and subjected to FACS (BD FACSAriaII) using immunostaining with follwing biotinylated antibodies (CD45, CD11b, CD31 and Sca1, eBioscience), streptavidin-APC-Cy7, CD34-Alexa Fluor 647 and Integrin α7- FITC (eBioscience). For the mitochondria staining, 200 nM Mito-Tracker Green (Beyotime) was added to muscle digests and incubated for 1 hour at 37ºC. For the intracellular staining, the SCs were firstly stained with the surface marker and then subjected to Fixation/Permeabilization Kit (BD) according to the manufacturer's instruction, and then the cells were stained with p-p38-PE (eBioscience), p-ERK-PE (Biolegend), p-Akt-FITC (Biolegend) or p-S6-PE (Biolegend). For the detection of H3 modification, the cells were stained with anti-H3K27me3 (CST), anti-H3K27ac (CST) or anti-H3K56ac (CST) and then incubated with anti-rabbit-FITC (Biolegend).

For BrdU incorporation analysis, SCs cultured in vitro were incubated with 10μM BrdU (Sigma) for 30 min, washed with PBS, fixed with 70% absolute ethyl alcohol for 6 hours, permeabilization with 0.1% Triton X-100 for 10 min, incubation with 2M HCl for 30 min, and blocked with 2% horse serum for 1 h. Then cells were stained with a BrdU specific antibody conjugated with FITC (eBioscience) for 30 min and analyzed by flow cytometry.

**Immunofluorescence**. Sections of TA, myofibers or SCs were fixed with 4% paraformaldehyde for 30 min at room temperature, washed and then incubated in 0.1% Trixon X-100 for 10 min. Then samples were blocked with 2% horse serum for 1 h and incubated with primary antibodies at 4℃ overnight. The following antibodies were used: Pax7 (Invitrogen, 1:100), MyoD (Santa, 1:100), Laminin (Sigma, 1:250) and MyHC (R&D, 1:50). After the primary antibody incubation, myofibers were incubated with secondary antibodies conjugated with Alexa Fluor 488 or 647, or Cy3 (Beyotime, 1:500). Finally, the nucleus was staining by DAPI (5μg/ml, Sigma) for 10 min and the fluorescence pictures were captured by confocal microscopy (Olympus). For SCs number counting, total 2×10^5^ cells were seeded in 12-well plates. The cells were fixed with 4% paraformaldehyde for 10 min at 6 hours or 48 hours, and incubated with DAPI (5μg/ml) for 10 min.

**Cell proliferation assay**. To assess the altered cell phenotypes, we seeded the SCs in 96-well plates with 2,000 cells per well in triplicate. At 24, 48 and 72 hours post culture, cell proliferation was assessed using the Cell Counting Kit-8 Kit (Beyotime) according to the manufacturer's instruction.

**Library preparation and RNA-Sequence.** About 800 SCs were isolated by FACS and the mRNA library constructed using QIAseq FX Single Cell RNA Library Kit (QIAGEN) according to the manufacturer's instruction. Libraries were sequenced by the Illumina HiSeq 2000 platform as 150-bp pair-ended reads. Reads were aligned using bowtie v0.12.9. FPKM estimation was performed with Cufflinks v2.1.1, aligned reads were counted with HTSeq, and differential expression analysis was performed with DESeq2. Differentially expressed genes were selected using a cut-off at a P value of less than 0.05 (FDR adjusted for multiple testing). For functional profiling of changes in mRNA by RNA-Sequence, GSEA analysis was performed using the GSEA software.

**RNA extraction and quantitative real time PCR.** Total cellular RNA was isolated from 3000 SCs immediately after FACS sorting using the Total RNA isolation Kit (Thermo Fisher) according to the manufacturer's instructions. For SCs cultured in vitro, 1 million SCs were used for RNA isolation. cDNA was reverse transcribed using PrimeScript RT reagent Kit (Takara) and subjected to real-time PCR with SYBR Green Supermix (Bio-Rad) in an iCycler iQ Real Time PCR Detection System (Bio-rad). All primers are listed in Table S1. All samples were run in triplicate. β-actin (Actinb) was used as an internal control for mRNA.

**Mitochondria DNA quantification**. Total DNA was isolated from 20,000 SCs immediately after FACS sorting using Hipure Tissue DNA Mini Kit (Magen) according to the manufacturer's instructions. mtDNA was analyzed by quantitative PCR. All primers are listed in Table S1.

**Glucose Consumption.** Glucose concentration in the medium was analyzed using the Amplex Red Glucose/Glucose Oxidase Assay Kit (Invitrogen).

**ATP and acetyl-CoA measurement.** 30,000 SCs were isolated by FACS and counted with a haemocytometer immediately after isolation. The cellular ATP level were measured using Enhanced ATP Assay Kit (Beyotime) according to the manufacturer's instructions. For SCs cultured *in vitro*, the intracellular levels of acetyl-CoA were measured using the PicoProbe Acetyl CoA Assay Kit (Abcam) according to the manufacturer's instructions.

**Protein Biogenesis Assay.** SCs were cultured in vitro, and the dilute Click-iT OPP (O-propargyl-puromycin) was added into the medium with a 20 μM working solution for 1 hour. Then the SCs were detected the incorporation of OP-puro using the Click-iT Plus OPP Protein Synthesis Assay Kit (Molecular Probes).

**Measurement of activities of mitochondrial respiratory chain complex.** Activities of mitochondrial respiratory chain complex I, II, and III were assessed by the corresponding assay kit (Solarbio).

**Western blot**. For western blot, the SCs cultured in vitro were extracted in RIPA lysis buffer (1× PBS, 1% NP-40, 0.1% SDS, 0.5% sodium deoxycholate, 1 mM EDTA). Protein extracts were subjected to electrophoresis on polyacrylamide gels and transferred to nitrocellulose membranes. The membranes were first incubated in blocking buffer and then incubated with antibodies. The antibodies used in this study were against: p-p38 (Beyotime, 1:1000), p38 (Beyotime, 1:1000), p-ERK (Beyotime, 1:1000), ERK (Beyotime, 1:1000), Tubulin (Beyotime, 1:1000), p-Akt (Beyotime, 1:1000), Akt (Beyotime, 1:1000), H3K4me1 (CST, 1:1000), H3K4me3 (CST, 1:1000), H3K9me3 (CST, 1:1000), H3K27me3 (CST, 1:1000), H3K9ac (CST, 1:1000), H3K18ac (CST, 1:1000), H3K27ac (CST, 1:1000), H3K56ac (CST, 1:1000), H3 (Beyotime, 1:1000). The HRP-conjugated secondary antibodies were Goat anti Rabbit and Goat anti mouse (Beyotime, 1:1000). Uncropped immunoblots are located in Supplementary Figure 4.

**Chromatin extraction and analysis**. 500,000 SCs cultured in vitro were harvested and resuspended in 1 ml nuclear extraction buffer (10 mM Tris-HCl (pH 7.4), 10mM NaCl, 3mM MgCl_2_, 0.1% NP-40) on ice for 10 min. Nuclei were collected by centrifugation and washed twice. Finally, nuclei were resuspended in 50 μl digestion buffer (15mM Tris-HCl (pH7.4), 1mM CaCl_2_) and digested with micrococcal nuclease (0.01U/ml, Thermo Fisher) for the indicated time. The reactions were stopped by adding 10 μl stop solution (2% SDS, 50 mM EDTA). The DNA was purified using Hipure Tissue DNA Mini Kit (Magen) and subject to agarose gel electrophoresis.

**ChIP assay**. SCs cultured in vitro were harvested and used for ChIP assays. ChIP assays were performed using EZ-ChIP Chromatin Immunoprecipitation Kit (Milipore). In brief, the cells were fixed with 1% formaldehyde for 10 min, and the fixation reaction was quenched with glycine to a final concentration of 125 mM. The cells were lysed and sonicated until the desired lengths were achieved (100-500 bp). Then, 5 μg of anti-H3K27ac (CST), anti-H3K56ac (CST), anti-H3K27me3 (CST) or control IgG were used for immunoprecipitation. After elution of DNA from precipitated immunocomplexes, PCR or quantitative real-time PCR were performed with specific primers (Table S1).

**Lentivitral constructs and packaging.** To generate the vectors for the expression of Igfbp7 specific shRNA, we designed the sequence of shRNAs and cloned shRNAs into the vector pLKO.1-puro (primer sequences, Table S1). For lentivirus production, 293T cells were transfected with the helper plasmid pSPAX2 and pMD2.G. The medium was replaced with fresh medium at 10 h after transfection. The culture supernatants were collected at 48 h after transfection and filtered by 0.22 μm membrane. Virus was stored at -80 ℃ until use. For lentiviral infection of cells in vitro, lentiviral stock was added to SCs with polybrene (8μg/ml, Sigma-Aldrich, USA). For lentiviral infection of cells in vivo, lentivirus-shIgfbp7 was injected in muscles twice.

**Statistical analysis**. All experiments included at least three biological replicates. Comparisons between two groups were analyzed using two-tailed Student's t-test. Differences among more than two groups were analyzed using one-way ANOVA followed by Tukey-Kramer post-hoc tests. Values of *p*<0.05 were considered statistically significant. All data are means ± SD.

**Accession numbers**. All Sequencing data are deposited in the NCBI GEO database under accession number: GSE128651 and GSE37525.

## Supplementary Material

Supplementary figures and tables.

## Abbreviations

SCs: muscle satellite cells
ERK: extracellular regulated protein kinase
MAPK: the mitogen-activated protein kinase
HE: Haematoxylin and eosin
DMD: Duchenne muscular dystrophy
AMPK: AMP-activated protein kinase
PPAR: Peroxisome proliferator-activator receptors
PGC-1α: Peroxisome proliferator-activator receptor-γ (PPARγ) coactivator-1α
TA: Tibialis anterior
EDL: Extensor digitorum longus
CSA: cross-sectional area
DPI: days post-injury
GSEA: Gene-set-enrichment analysis
TCA: Tricarboxylic acid
Igfbps: Insulin-like growth factor-binding proteins (Igfbps)
H3: Histone 3
H3K27me3: Triple methylated H3K27
H3K27ac: Acetylated H3K27
H3K56ac: Acetylated H3K56
Acly: ATP citrate lyase
