# Supplementary material for: Exercise protects proliferative muscle satellite cells against exhaustion via the Igfbp7-Akt-mTOR axis
Source: Theranostics. 2020 May 16;10(14):6448–66. doi: 10.7150/thno.43577 (PMC7255041; doi:10.7150/thno.43577)
Supplement: Supplementary file 1 — Supplementary figures and tables. [file thnov10p6448s1.pdf]

# Supplementary Figures and Figure legends

## Figure S1

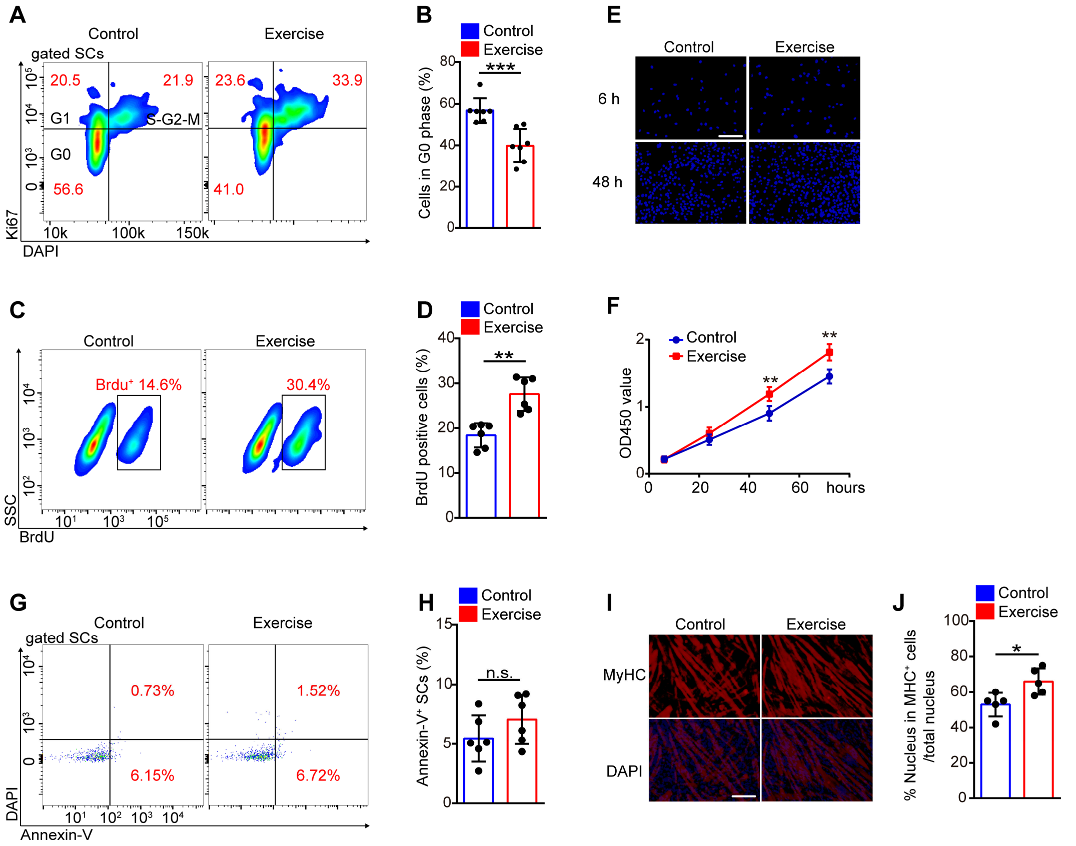


**Supplementary Figure 1. The effect of exercise on proliferation, apoptosis, and differentiation of SCs**

(**A**) The mononuclear cells isolated from skeletal muscles were stained with cell surface markers, DAPI and Ki67. The quiescent percentage of SCs was analyzed by flow cytometry. (**B**) Quantification of the percentage of G0 phase in SCs (n=7 mice). (**C**) SCs were isolated from mice and cultured *in vitro*. SCs were labeled with BrdU. Flow cytometry analysis was conducted to detected BrdU$^+$ cells. (**D**) The frequency of BrdU$^+$ SCs (n=6). **(E)** Representative fluorescent images of immunostaining for SCs cultured in *vitro*. SCs were stained with DAPI. Scale bars, 100 µm. (**F**) *In vitro* proliferation assay (CCK8) of cultured SCs (n=5). (**G**) The mononuclear cells isolated from skeletal muscles in mice were stained with SC markers, as well as Annexin-V and DAPI. The population of apoptotic SCs was analyzed by flow cytometry. (**H**) The percentage of apoptotic SCs (Annexin-V$^+$ SCs) (n=6 mice). (**I**) SCs were isolated from mice and cultured in differentiation medium for 72 hours. Immunofluorescence of MHC in SCs. Nucleus was stained with DAPI. Scale bar, 100µm. (**J**) Quantification the percentage of nucleus in MHC$^+$ cells/total cells (n=5). Error bars represent the means ± SD. *$p$<0.05, **$p$<0.01, ***$p$<0.001; n.s. no significance; Student's $t$-test.

## Figure S2

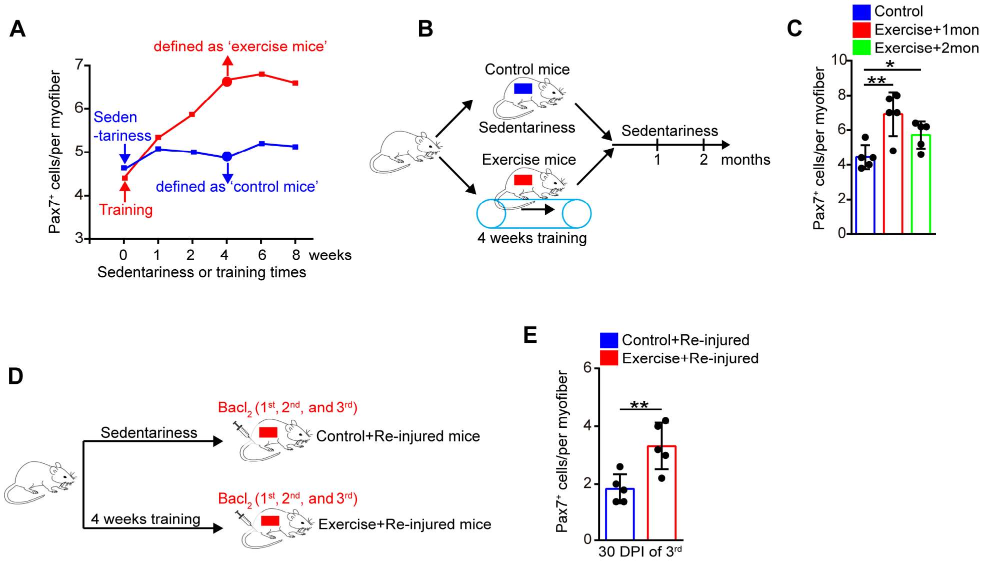


**Supplementary Figure 2. The effect of exercise on SC maintenance**

**(A)** The mice were subjected to sedentariness or training. Skeletal muscles were harvested depending on sedentariness or training time (0, 1, 2, 4, 6 and 8 weeks) and digested being single myofiber. Myofibers were fixed and stained by the Pax7 antibody, and the numbers of Pax7$^+$ SCs per myofiber were counted (n=3 mice, 20 myofibers per mouse). (**B**) A schematic illustration showing the design for the experiment. Sedentary mice defined as 'control mice'. Mice were trained for 4 weeks and then subjected to sedentariness for 1 month (defined as 'exercise+1mon') or 2 months (defined as 'exercise+2mon'). (**C**) Quantification of Pax7$^+$ SCs number per myofiber in mice (n=5 mice). (**D**) A schematic illustration showing the design for repeated muscle injuries (three times, interval for 14 days, repeated injured was defined as "Re-injured") (E) Quantification of Pax7$^+$ SCs number per myofiber of mice at 30 days post-injury (3$^{rd}$ ) (n=5 mice, 20 myofibers per mouse). Error bars represent the means ± SD. **p*<0.05, ***p*<0.01; One-way ANOVA.

**Figure S3**

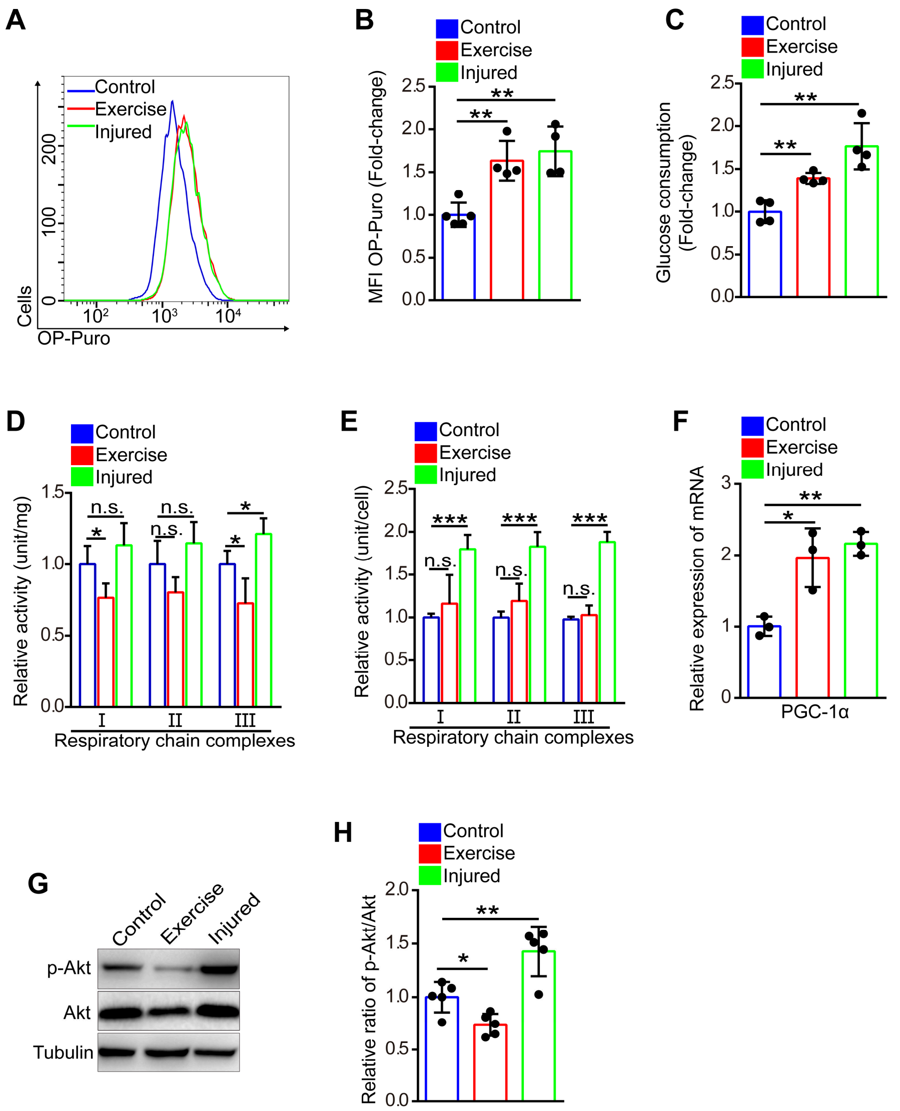


**Supplementary Figure 3. The effect of exercise on SC metabolism and phosphorylation of Akt**

(**A**) SCs were isolated from mice and cultured *in vitro*, the OP-Puro was added to the culture medium for 1 hour. Flow cytometry analysis was conducted to detect the intensity of OP-Puro. (**B**) The MFI analysis of OP-Puro in SCs (n=4). (**C**) SCs were isolated from mice and cultured for 24 hours *in vitro***,** the change of glucose concentration in the culture medium was measured (n=4). (**D**, **E**) SCs were isolated from mice and cultured *in vitro*, the activity of respiratory chain complex was quantified as unit per mg protein (**D**), or unit per cell (**E**) (n=4). (**F**) SCs were sorted from mice. Total RNA of SCs was extracted and the expression of PGC-1α was assayed by qPCR (n=3 mice). (**G**) SCs were isolated from mice and cultured *in vitro*. The protein levels of p-Akt, Akt and Tubulin (loading control) were analyzed by western blot (n=3). Error bars represent the means ± SD. **p*<0.05, ***p*<0.01, ****p*<0.001; n.s. no significance; One-way ANOVA.

## Figure S4

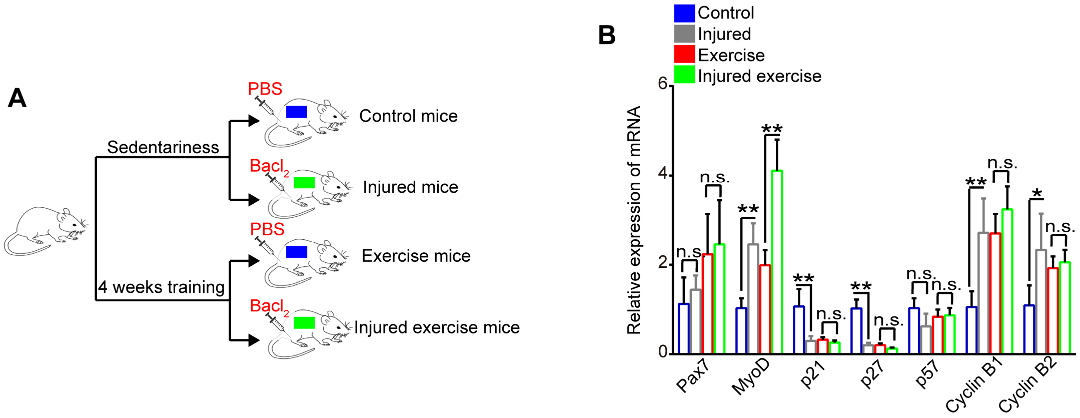


**Supplementary Figure 4. The effect of exercise on gene expression of SCs**

(**A**) A schematic illustration showing the design for muscle injury. Mice were subjected to sedentariness or training for 4 weeks, then PBS or $Bacl_2$ were injected into mice. (**B**) SCs were sorted from mice at 48 hours post-injection. Total RNA of SCs was extracted and the expression of indicated genes was assayed by qPCR (n=4 mice). Error bars represent the means ± SD. *$p<0.05$, **$p<0.01$; n.s. no significance; One-way ANOVA.

## Figure S5

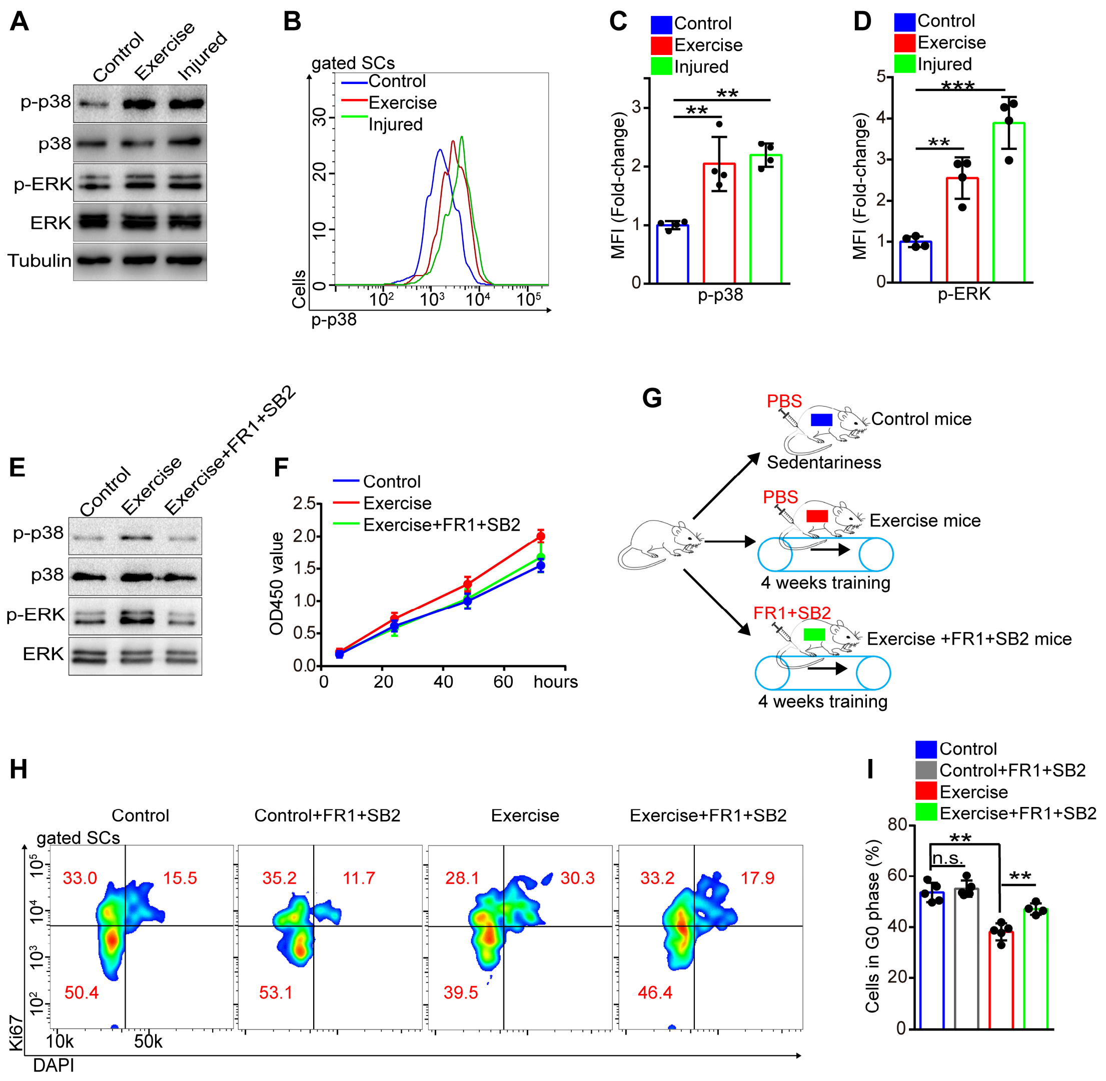


**Supplementary Figure 5. Exercise promotes SCs cell cycling by MAPK pathway**

(A) SCs were isolated from mice and cultured *in vitro*. The protein levels of p-p38, p38, p-ERK, ERK and Tubulin (loading control) were analyzed by western blot (n=3). (B) The mononuclear cells isolated from skeletal muscles were stained with SC markers and p-p38 fluorescent antibody. The intensity of p-p38 was analyzed by flow cytometry. (**C**) The MFI analysis of p-p38 in SCs in mice (n=4 mice). (**D**) The MFI analysis of p-ERK in SCs in mice (n=4 mice). (**E**) SCs isolated from control and exercise mice were cultured *in vitro* with the treatment of inhibitors of p38 (SB203580, SB2) and ERK (FR180204, FR1) for 24 hours. The protein levels of p-p38, p38, p-ERK, and ERK were analyzed by western blot (n=3). (**F**) *In vitro* proliferation assay (CCK8) of cultured SCs (n=5). (**G**) A schematic illustration showing the design for the treatment of FR1 and SB2. Sedentary mice were defined as “control mice” that injected with PBS. For experimental preciseness, “exercise mice” received PBS injection, and mice were defined as “exercise+FR1+SB2 mice” that subjected to 4 weeks of training and synchronized FR1 and SB2 injection. (**H**) The mononuclear cells isolated from skeletal muscles were stained with SC markers,

DAPI and Ki67. (**I**) Quantification of the percentage of G0 phase in SCs (n=4-5 mice). Error bars represent the means ± SD. *$p$<0.05, **$p$<0.01, ***$p$<0.001; n.s. no significance; One-way ANOVA.

## Figure S6

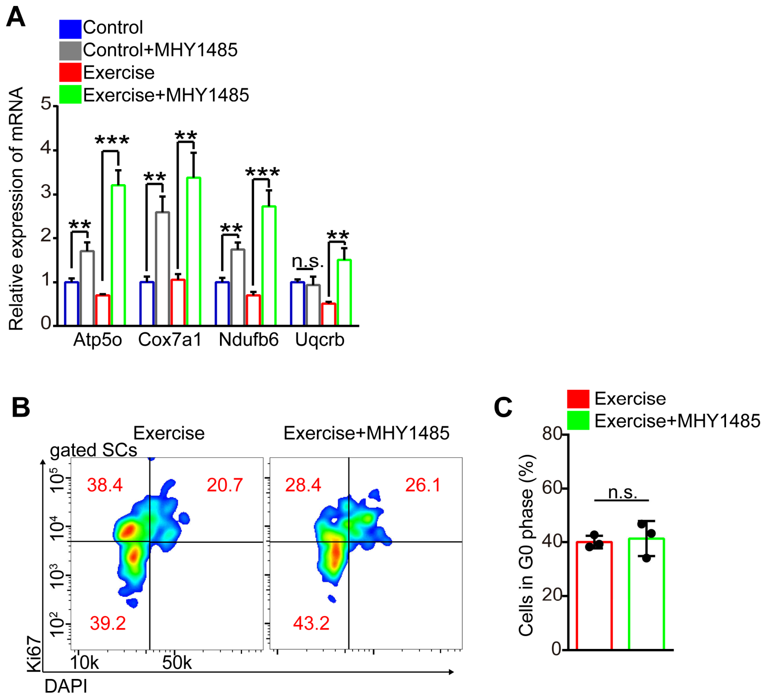


**Supplementary Figure 6. The effect of MHY1485 on gene expression and cell cycle of SCs**

(**A**) SCs were sorted from mice with or without MHY1485 treatment. Total RNA of SCs was extracted and the expression of indicated genes was assayed by qPCR (n=4 mice). (**B**) The mononuclear cells isolated from skeletal muscles were stained with SC markers, DAPI and Ki67. (**C**) Quantification of the percentage of G0 phase in SCs (n=3 mice). Error bars represent the means ± SD. $^{**}p<0.01$, $^{***}p<0.001$; n.s. no significance; One-way ANOVA.

## Figure S7

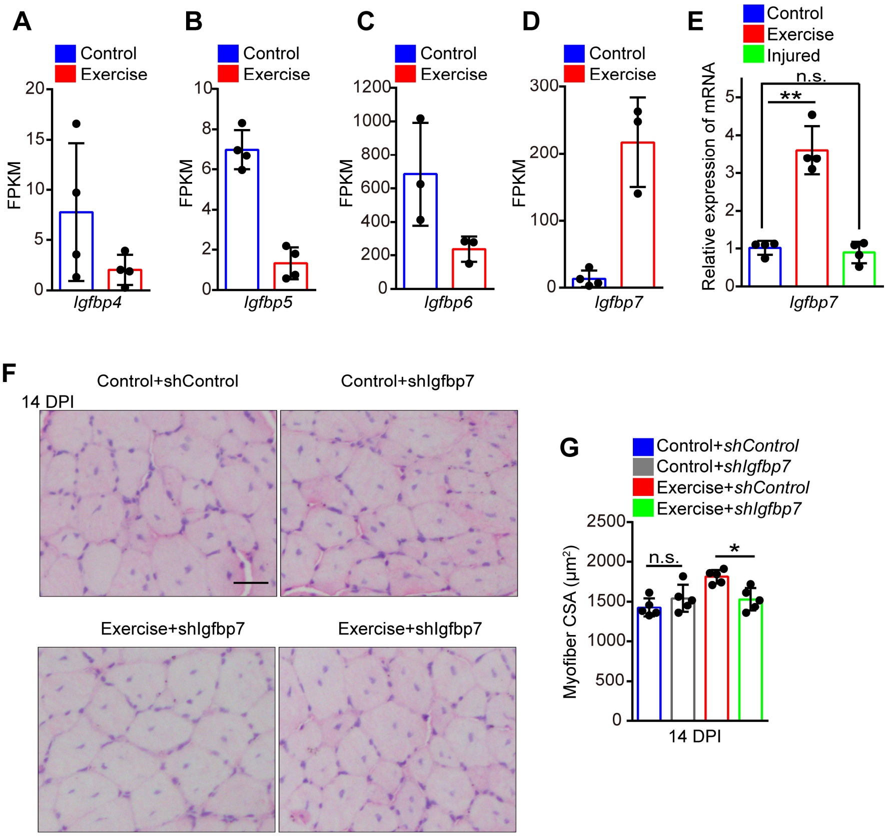


**Supplementary Figure 7. The effect of knock-down of Igfbp7 in SCs on muscle regeneration**

(**A-D**) The reads per kilobase per million (FPKM) value of Igfbp4, Igfbp5, Igfbp6, and Igfbp7 of SCs (n=3-4 mice, data from RNA-Sequence). (**E**) SCs were sorted from mice. Total RNA of SCs was extracted and the expression of Igfbp7 was assayed by qPCR (n=4 mice). (**F**) HE staining of the cross-sections of TA of control and exercise mice at 14 DPI. Scale bar, 100μm. (**G**) Average CSA of TA of control and exercise mice at 14 DPI (n=5 mice). Error bars represent the means ± SD. *$p$<0.05, **$p$<0.01; n.s. no significance; One-way ANOVA.

## Figure S8

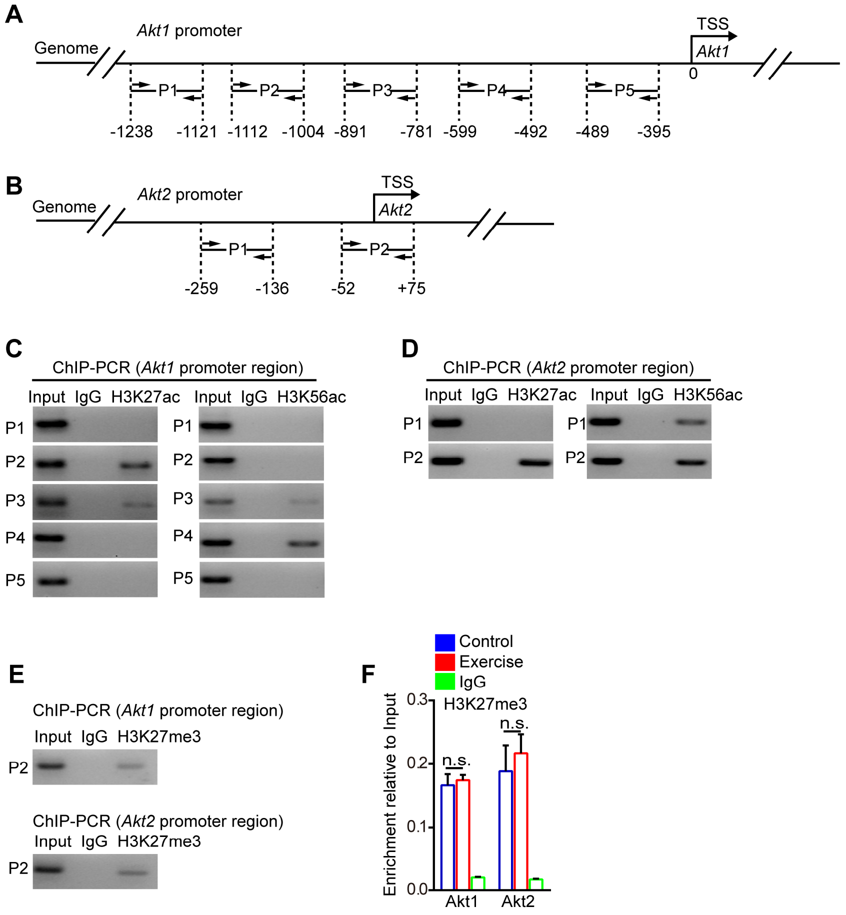


**Supplementary Figure 8. ChIP-PCR analysis of H3K27ac and H3K56ac at promoters regions of Akt**

(**A**, **B**) Schematic diagram of the upstream promoter regions of the Akt1 and Akt2 genes. Potential binding regions of H3K27ac and H3K56ac are shown: P1 (−1238 ~ −1121), P2 (−1112 ~ −1004), P3 (−891 ~ −781), P4 (−599~−492), and P5 (−489~−395) in Akt1 promoter region (**A**); and P1 (−259 ~ −136) and P2 (−52 ~ +75) in Akt2 promoter region (**B**). (**C**, **D**) Endogenous binding of H3K27ac and H3K56ac to the promoter regions of Akt1 (**C**) and Akt2 (**D**). IgG was used as a negative control. (**E**) Endogenous binding of H3K27me3 to the promoter regions of Akt1 and Akt2. (**F**) SCs were isolated and cultured *in vitro*. Quantitative ChIP-PCR was utilized to detect the binding of H3K27me3 at promoter regions of Akt1/2 in SCs. IgG served as a negative control. Enrichment relative to the input was shown (n = 4). Error bars represent the means ± SD. n.s. no significance; One-way ANOVA.

## Figure S9

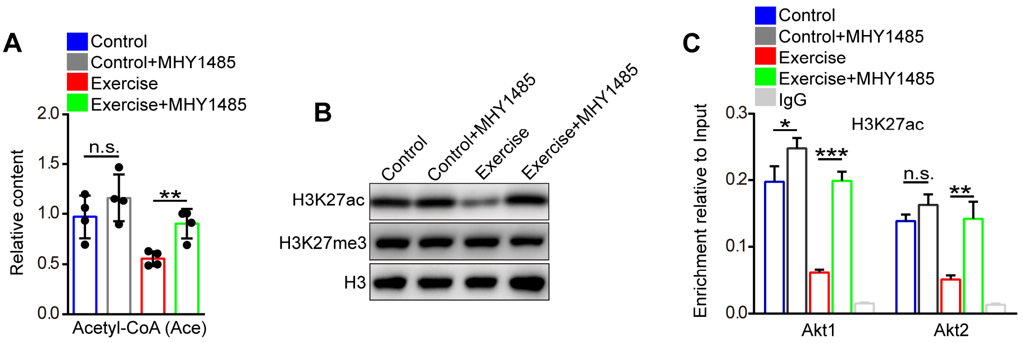


**Supplementary Figure 9. The effect of MHY1485 treatment on acetylation of H3K27**

**(A)** SCs were isolated from mice with or without MHY1485 treatment and cultured *in vitro*. The intracellular acetyl-CoA (Ace) levels of SCs were analyzed (n=4). (**B**) SCs were isolated from mice and cultured *in vitro*. The protein levels of H3K27ac, H3K27me3 and H3 (loading control) were analyzed by western blot (n=3). (**C**) SCs were isolated and cultured *in vitro*. Quantitative ChIP-PCR was utilized to detect the binding of H3K27ac at promoter regions of Akt1/2 in SCs. IgG served as a negative control. Enrichment relative to the input was shown (n = 4). Error bars represent the means ± SD. **p*<0.05, ***p*<0.01, ****p*<0.001; n.s. no significance; One-way ANOVA.

## Figure S10

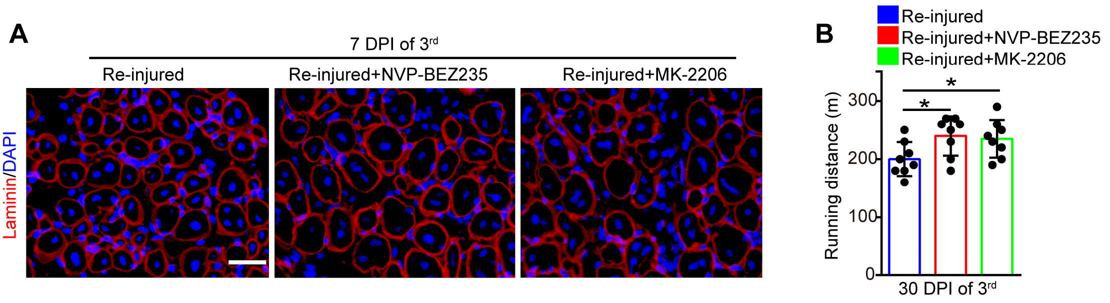


**Supplementary Figure 10. The effect of NVP-BEZ235 and MK-2206 on muscle regeneration**

**(A)** Immunofluorescence of Laminin and DAPI on cross-sections of TA of mice at 7 DPI of 3rd injury. Scale bars, 100 μm. (**B**) Mice were acclimated to running on the treadmill until exhaustion. The maximum running distances of mice at 30 DPI of 3rd injury were measured (n=8 mice). Error bars represent the means ± SD. *$p$<0.05; One-way ANOVA.

# Figure S11

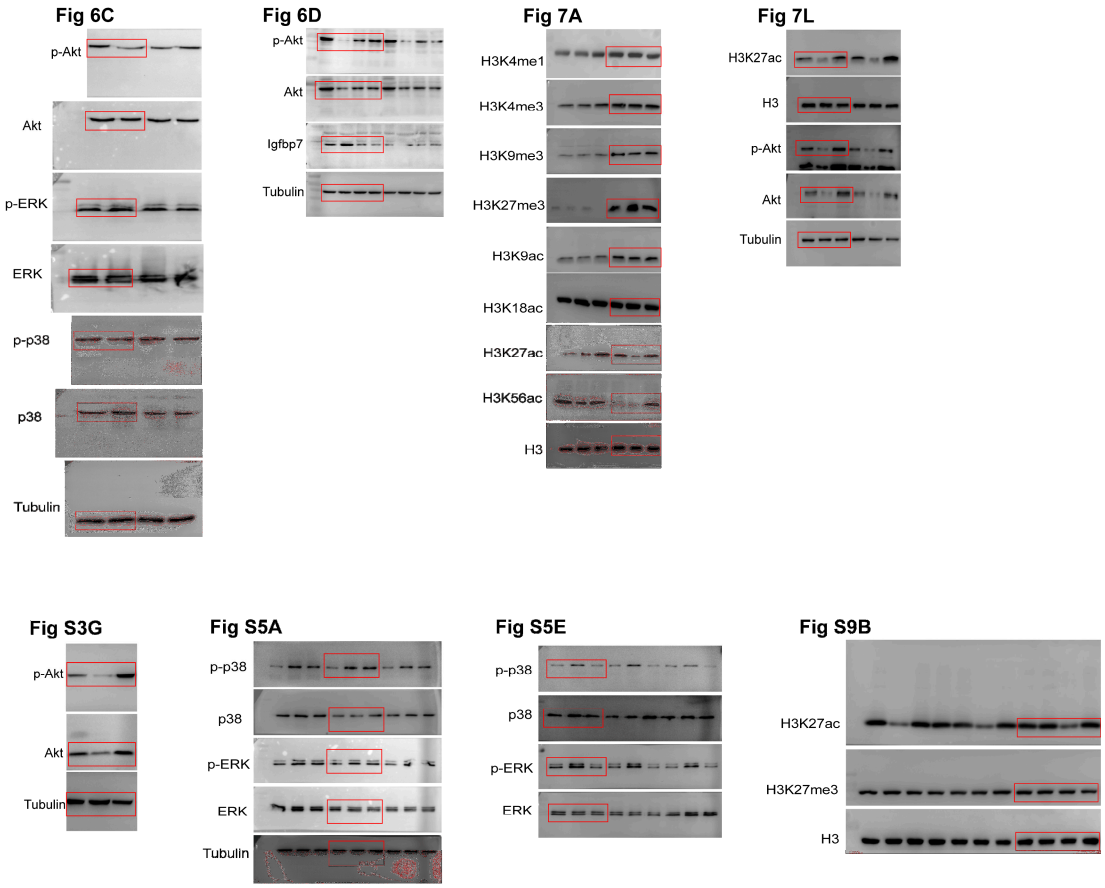


**Supplementary Figure 11. Uncropped figures of western blot**

# Supplementary Table

**Table S1: The sequences of primers**

| Primers for RT-PCR | |
|---|---|
| Pax7-F | GCTACCAGTACAGCCAGTATG |
| Pax7-R | GTAGGCTTGTCCCGTTTCC |
| MyoD-F | CGCTCCAACTGCTCTGATG |
| MyoD-R | ACACAGCCGCACTCTTC |
| P16-F | CTCTGGCTTTCGTGAACATG |
| P16-R | TCGAATCTGCACCGTAGTTG |
| P21-F | CTTGCACTCTGGTGTCTGAG |
| P21-R | GCACTTCAGGGTTTTCTCTTG |
| P27-F | TGGACCAAATGCCTGACTC |
| P27-R | GGGAACCGTCTGAAACATTTTC |
| P57-F | CAGGACGAGAATCAAGAGCAG |
| P57-R | CGACGCCTTGTTCTCCTG |
| Cyclin B1-F | CTGACCCAAACCTCTGTAGTG |
| Cyclin B1-R | CCTGTATTAGCCAGTCAATGAGG |
| Cyclin B2-F | CCTCAGAACACCAAAGTACCAG |
| Cyclin B2-R | CCTTCATGGAGACATCCTCAG |
| Acly-F | AAGCCTACATTGCAGACCTG |
| Acly-R | TTGACACCTCCAAGATCACAG |
| Hagh-F | TGGGCTGAAGGTTTATGGAG |
| Hagh-R | CAGATGTGTCCCGAAGTATGG |
| Grhpr-F | AGGTGGAACAGTGGAATTCG |
| Grhpr-R | GATGACTCTGAGGTTGGCTC |
| Ldha-F | ATGAAGGACTTGGCGGATG |
| Ldha-R | TGGAGTTCGCAGTTACACAG |
| Ldhd-F | ACGCTTTTCCTGGAGTTCC |
| Ldhd-R | CGCTGCCCAAAGTTCATTG |
| Acpy2-F | GGAGAAGCTAAGAAACGAGGTC |
| Acpy2-R | GTCGATGCGAGAACTAGGAC |
| Acaca-F | AAGGCTATGTGAAGGATGTGG |
| Acaca-R | CTGTCTGAAGAGGTTAGGGAAG |
| Idh3a-F | ACAAGACCCATCCCAGTTTG |
| Idh3a-R | GATTCAAAGATGGCAACACCG |
| Idh3b-F | GGAATATAGCCAACCCCACAG |
| Idh3b-R | TGTAGCCTCCCATGTCTCG |
| Idh3g-F | AGTGGGCGTAAGAAAGTGAC |

| | |
|---|---|
| Idh3g-R | CAAAGGTGATCTGAGGGTAGTG |
| Sdha-F | GATTACTCCAAGCCCATCCAG |
| Sdha-R | ACAGTCAGCCTCATTCAAGG |
| Sdhc-F | GTCTCTTCCTATGGCACTGTC |
| Sdhc-R | CAAACACAGGGACTTCACAAAC |
| Sucla2-F | TCCAGCAAGTAACAGAAGCG |
| Sucla2-R | ACTGCCATGACTATTCCTTGTG |
| Fh1-F | ATATTCGCTTCCTGGGTTCTG |
| Fh1-R | GTCATCGCTTCACACTGAGTAG |
| Pten-F | TGTAAAGCTGGAAAGGGACG |
| Pten-R | CCTCTGACTGGGAATTGTGAC |
| Akt1-F | GCCCTCAAGTACTCATTCCAG |
| Akt1-R | ACACAATCTCCGCACCATAG |
| Akt2-F | TTACAGCCCTCAAGTATGCC |
| Akt2-R | GACACAATCTCTGCTCCATAAAAG |
| Rptor-F | CACTCCTTGTCTTCATCTGGG |
| Rptor-R | TGTCATGGTCCTATGTTCAGC |
| Rictor-F | ATGGAAATAAGGCGAGGTCTG |
| Rictor-R | AAAGCCTCCAACTGTCCTG |
| Atp5o-F | TCATCTCTGCCTTTTCCACC |
| Atp5o-R | TGGTTTGGACTCAGGAAGC |
| Cox7a1-F | TGAGGACGCAAAATGAGGG |
| Cox7a1-R | CTTTCAAGTGTACTGGGAGGTC |
| Ndufb6-F | CTTCGCTGTTTCTCATGTGC |
| Ndufb6-R | CCAGTCTCCAGAATTGTATCACC |
| Uqcrb-F | GACTTACCCAGAAGGCAGC |
| Uqcrb-R | CATCTCGCATTAACCCCAGT |
| PGC-1α-F | CACCAAACCCACAGAAAACAG |
| PGC-1α-R | GGGTCAGAGGAAGAGATAAAGTTG |
| β-Actin-F | ACCTTCTACAATGAGCTGCG |
| β-Actin-R | CTGGATGGCTACGTACATGG |
| Primers for mitochondria DNA quantification | |
| mt DNA qPCR primer-F | CATTTATTATCGCGGCCCTA |
| mt DNA qPCR primer-R | TGTTGGGTTGTTTGATCCTG |
| β-globin gDNA qPCR -F | GAAGCGATTCTAGGGAGCAG |
| β-globin gDNA | GGAGCAGCGATTCTGAGTAGA |

| | |
|---|---|
| qPCR -R | |
| Primers for ChIP-PCR | |
| Akt1-P1 region-F | GAGCATCCGAGTGAGAAG |
| Akt1-P1 region-R | CTCCTCCTCTCCTACTTCC |
| Akt1-P2 region-F | AGTAGGAGAGGAGGAGTTG |
| Akt1-P2 region-R | CAGCAGCAGTTCTGTTCT |
| Akt1-P3 region-F | CTGAGAGGAGAGTGAGTTC |
| Akt1-P3 region-R | TTAGTAGTCCACGCTATCG |
| Akt1-P4 region-F | AGGACTGAGGATGGATAAGA |
| Akt1-P4 region-R | AAGACCTTGCTGTGGACT |
| Akt1-P5 region-F | AGTCCACAGCAAGGTCTT |
| Akt1-P5 region-R | CCACAGCAACTGATAAGGA |
| Akt2-P1 region-F | GGAACTAGGAAGGCAGGA |
| Akt2-P1 region-R | CAGTAGGACACCAACAAGTA |
| Akt2-P2 region-F | TGACGGGTGCCTAAAGTA |
| Akt2-P2 region-R | CAGCCACAAACAGGAACT |
| GAPDH ChIP-F | CAAGGAGCCAAGACTAGATT |
| GAPDH ChIP-R | TCAAGAGCCTATTGCTAAGT |
| Primers used for subclone | |
| mIgfbp7 shRNA-1-F | CCGGCCTCCATGAAATACCACTGAACTCGAGTTCAGTGGTATTTCATGGAGGTTTTTG |
| mIgfbp7 shRNA-1-R | AATTCAAAAACCTCCATGAAATACCACTGAACTCGAGTTCAGTGGTATTTCATGGAGG |
| mIgfbp7 shRNA-2-F | CCGGCCTCATCTGGAACAAGGTAAACTCGAGTTTACCTTGTTCCAGATGAGGTTTTTG |
| mIgfbp7 shRNA-2-R | AATTCAAAAACCTCATCTGGAACAAGGTAAACTCGAGTTTACCTTGTTCCAGATGAGG |
